# Structure-Activity Relationship between Thiol Group-Trapping Ability of Morphinan Compounds with a Michael Acceptor and Anti-*Plasmodium falciparum* Activities

**DOI:** 10.3390/molecules25051112

**Published:** 2020-03-02

**Authors:** Noriki Kutsumura, Yasuaki Koyama, Tsuyoshi Saitoh, Naoshi Yamamoto, Yasuyuki Nagumo, Yoshiyuki Miyata, Rei Hokari, Aki Ishiyama, Masato Iwatsuki, Kazuhiko Otoguro, Satoshi Ōmura, Hiroshi Nagase

**Affiliations:** 1 International Institute for Integrative Sleep Medicine (WPI-IIIS), University of Tsukuba, 1-1-1 Tennodai, Tsukuba, Ibaraki 305-8575, Japan; kutsumura.noriki.gn@u.tsukuba.ac.jp (N.K.); tsuyoshi-saito.gf@u.tsukuba.ac.jp (T.S.); yamamoto.naoshi.gu@u.tsukuba.ac.jp (N.Y.); nagumo.yasuyuki.fu@u.tsukuba.ac.jp (Y.N.); 2 Graduate School of Pure and Applied Sciences, University of Tsukuba, 1-1-1 Tennodai, Tsukuba, Ibaraki 305-8571, Japan; kykoyamayasuaki@gmail.com; 3 School of Medicine, Keio University, 35, Shinanomachi, Shinjuku, Tokyo 160-8582, Japan; yomiyat@keio.jp; 4 Kitasato Institute for Life Sciences, Kitasato University, 5-9-1 Shirokane, Minato-ku, Tokyo 108-8641, Japan; hokari@lisci.kitasato-u.ac.jp (R.H.); ishiyama@lisci.kitasato-u.ac.jp (A.I.); iwatuki@lisci.kitasato-u.ac.jp (M.I.); tsuchikurekai@gmail.com (K.O.); omuras@insti.kitasato-u.ac.jp (S.Ō.)

**Keywords:** morphinan, BNTX, δ opioid receptor antagonist, ^1^H-NMR experiments, mechanism elucidation

## Abstract

7-Benzylidenenaltrexone (BNTX) and most of its derivatives showed in vitro antimalarial activities against chloroquine-resistant and -sensitive *Plasmodium falciparum* strains (K1 and FCR3, respectively). In addition, the time-dependent changes of the addition reactions of the BNTX derivatives with 1-propanethiol were examined by ^1^H-NMR experiments to estimate their thiol group-trapping ability. The relative chemical reactivity of the BNTX derivatives to trap the thiol group of 1-propanethiol was correlated highly with the antimalarial activity. Therefore, the measurements of the thiol group-trapping ability of the BNTX derivatives with a Michael acceptor is expected to become an alternative method for in vitro malarial activity and related assays.

## 1. Introduction

Malaria is one of the world’s deadliest infectious diseases and it is widespread in the tropical and subtropical regions located in a broad band around the equator, including Africa, Southeast Asia, Middle East, and Latin America. In 2017, an estimated 219 million malaria infections were reported in 87 countries, including about 435,000 deaths from malaria [1]. Malaria also causes serious complications, such as cerebral malaria involving encephalopathy [2], blackwater fever [3], and acute respiratory distress syndrome (ARDS) [4]. Since 2001, the World Health Organization (WHO) has recommended Artemisinin-based combination therapy (ACT) for antimalarial medication. However, not only general drug-resistant type of malaria, such as chloroquine-resistance (CQ-resistance), but also artemisinin-resistant malaria has been identified, creating a critical social situation [5,6].

**Figure 1:**
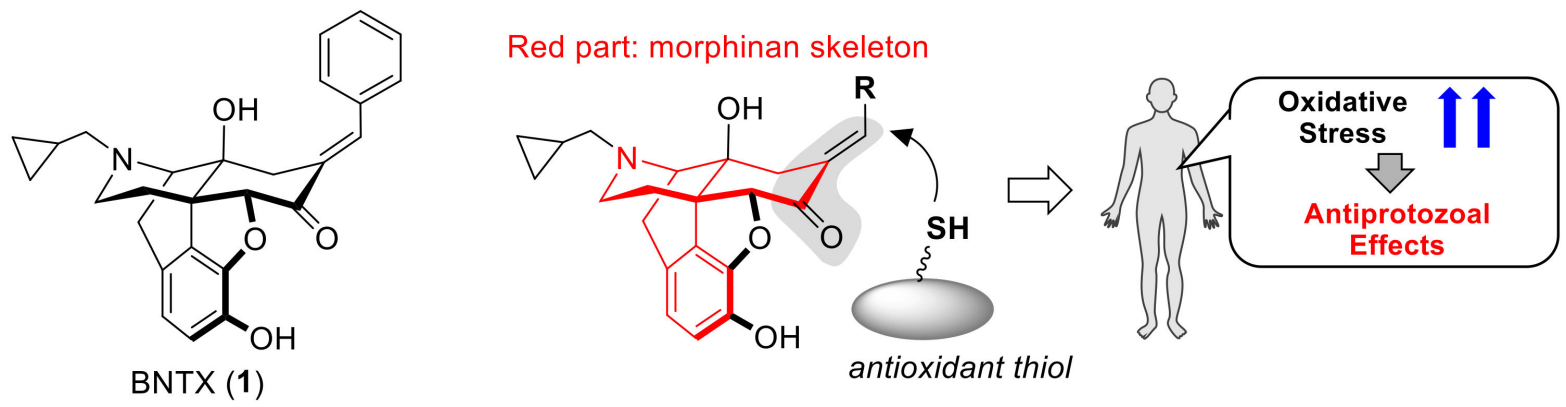
Structure of BNTX (**1**) and a plausible explication of antiprotozoal effects by the morphinan compounds.

Against such a background, we first reported that a δ opioid receptor (DOR) antagonist 7-benzylidenenaltrexone (BNTX, **1**, Figure 1) had a potent CQ-resistance reversing effect on *Plasmodium chabaudi*, that is, the combined administration of **1** (perorally) and CQ (intravenously) decreased the number of parasitized red blood cells in mice infected with CQ-resistant malaria [7]. These research results revealed that DOR antagonism was particularly important for the CQ-resistance reversing effect. Further studies suggested that the CQ-resistance reversing effect of **1** was closely related to not only the DOR antagonism but also to the presence of a Michael acceptor moiety in morphinan compound **1**. Although a correlation between the DOR antagonism and its CQ-resistance reversing effect on malaria parasites has not yet been clearly elucidated, the Michael acceptor structure could be involved in the effect by reacting with the thiol group of the glutathione-system to increase oxidative stress in the host [8]. In addition, Asahi and co-workers also reported that the morphinan **1** effectively inhibited the successive ring–trophozoite–schizont progression of *P. falciparum* during early developmental stages, which are associated with the development of pyknosis in ring forms, as compared to another antimalarial drug dihydroartemisinin [9]. In another study of **1** in protozoal infections, **1** and its derivatives exhibited in vitro antitrichomonal activity against *Trichomonas vaginalis* and the activity was also related to the presence of a Michael acceptor moiety in the morphinan derivatives [10,11]. Thus, “thiol group-trapping action by Michael acceptors such as an α,β-unsaturated ketone moiety” in morphinan compounds could be considered as a common factor for both the CQ-resistance reversing effect in malaria and the antitrichomonal effects. Therefore, we hypothesized that chemical enhancement of the ability to trap thiol groups (for example: glutathione et al. in malaria [12,13,14]; cysteine et al. in trichomonads [15,16,17,18,19]) would affect the inhibition of each antioxidant system, leading to the direct improvement of antiprotozoal activity (Figure 1). Morphinan compounds such as **1** have been recognized as structurally typical drug-like compounds, such as some compounds (morphine, codeine, and nalfurafine [20], etc.) have been used in clinical practice. In fact, we have already confirmed in vivo experiments that the morphinan compound **1** is effective against CQ-resistant malaria [7,8]. However, there have been no applications of morphinan compounds to protozoal infections, and this study is quite significant in the search for new lead compounds for protozoal infections. In this paper, we investigated the correlation between the thiol group-trapping ability of **1** and its derivatives and in vitro antimalarial activity.

## 2. Results and Discussion

First, BNTX (**1**) and the related derivatives **2**–**20** (see Table 1), which were prepared by the previously reported synthetic methods [10,11], were evaluated for in vitro antimalarial activities against CQ-resistant and -sensitive *Plasmodium falciparum* strains (K1 and FCR3, respectively) (Table 1) [21]. All compounds **1**–**20** exhibited moderate antimalarial activity against the CQ-resistant K1 strain (IC_50_ = 2.08–19.8 μM), except for the dimethylamino-substituted derivative **17**. These compounds also exhibited similar activity against the CQ-sensitive FCR3 strain (IC_50_ = 1.94–15.0 μM), with the exception of **17** and the reduced derivative **20**. Notably, the morphinan derivatives bearing an electron-withdrawing substituted benzylidene group such as the compounds **6**, **8**, **9**, and **11** tended to exhibit relatively high antimalarial activities. On the contrary, the electron-donating substituted derivatives **16** and **17** tended to exhibit weak activities. These results thus suggested that inductive effects caused by introducing substituents into the benzylidene site affected antimalarial activity to some extent. For the compounds with alkylidene groups (**14**, **15**, and **18**), the antimalarial activity increased as the ring size increased. Of particular importance is the result that the antimalarial activity of the saturated derivative **20** lacking a Michael acceptor was significantly deactivated, as in our previous studies [8,11]. In the first in vitro antimalarial activity evaluation of a variety of BNTX derivatives, almost all derivatives were found to exhibit moderate antimalarial activity, although none of them were as potent as the clinical drugs artemisinin and chloroquine. Furthermore, the antimalarial activity correlated with electron density of the Michael acceptor, as we predicted.

**Scheme 1:**
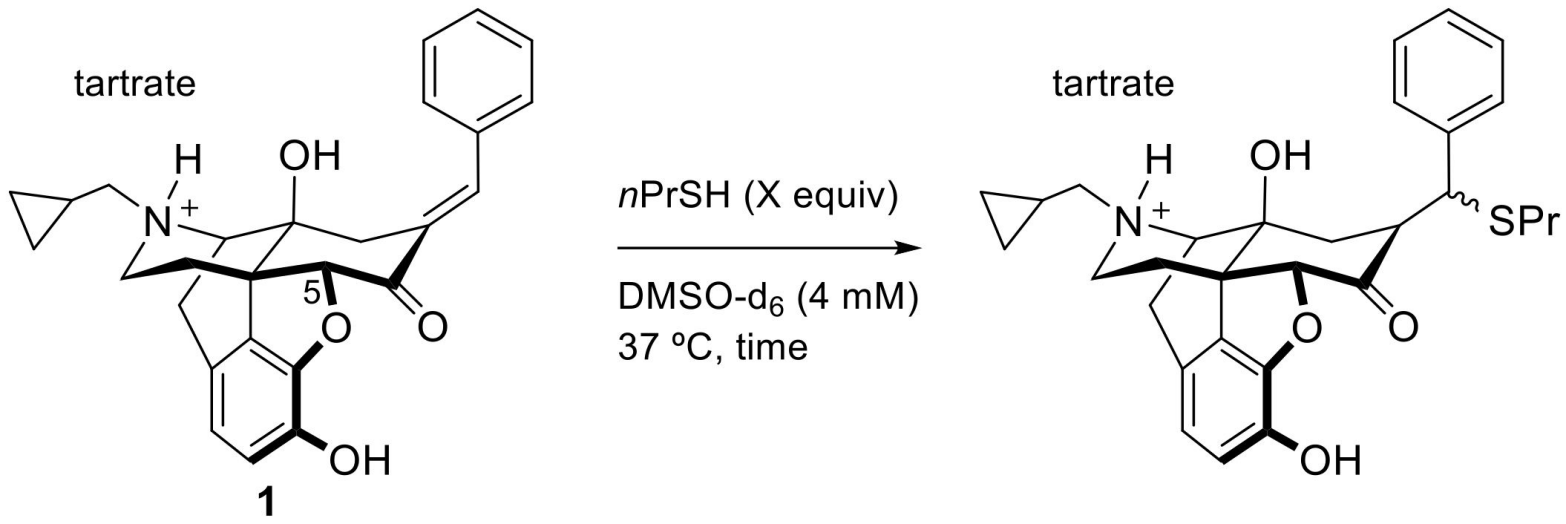
Reactions of **1** with 1-propanethiol.

**Figure 2:**
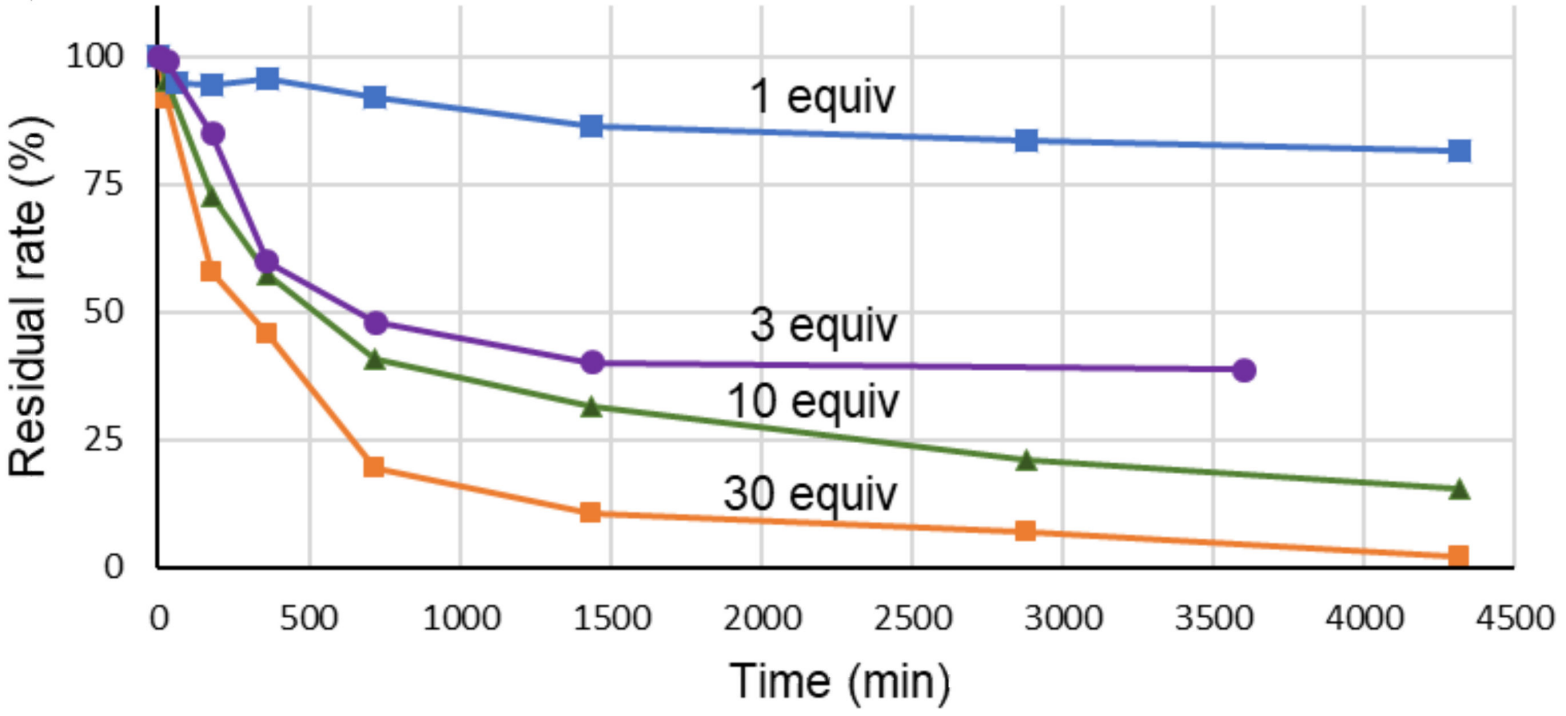
Temporal change of the addition reaction and the residual rate of **1**.

Next, to easily evaluate the thiol group-trapping ability of BNTX (**1**) and the various derivatives **2**–**20**, we examined the time-dependent changes of the addition reactions of the compounds **1**–**20** with 1-propanethiol (as a simple model compound with a thiol group) by utilizing ^1^H-NMR. A typical experimental example is as follows (Scheme 1). 1-Propanethiol was added to a solution of **1** in DMSO-*d*_6_ (4 mM) at 37 ⁰C, and the reaction system was kept at the same temperature. Then, the residual rate of **1** was calculated by measuring the integral value of the vinyl proton at constant time intervals. In addition, the time-dependent change of each reaction of **1** with the addition of increasing amounts of 1-propanethiol was performed (1, 3, 10, and 30 equiv.) as shown in Figure 2.

The correlation chart between the elapsed time of the reaction and the residual rate of **1** showed that the addition reaction of 1-propanethiol achieved equilibrium and the residual rate of substrate **1** converged to a steady-state. General glutathione levels in healthy human erythrocytes cover a wide range (0.4 to 3.0 mM [22] or 0.1 to 10 mM [23]). Therefore, we decided to evaluate the thiol group-trapping ability of **1**–**20** with 3 equivalents of 1-propanethiol (12 mM), because the addition reaction proceeded very slowly when the concentration of 1-propanethiol was 4.0 mM (1.0 equivalent) as shown in Figure 2. Table 2 shows the residual rate (%) of the starting materials **1**–**20** after 1 day or 2 days (also see the Supplementary Materials). The residual rate of starting morphinan derivatives was calculated by measuring the integral value of the proton at the C5-posidion because of ease of comparison of integral values before and after addition reaction.

**Figure 3:**
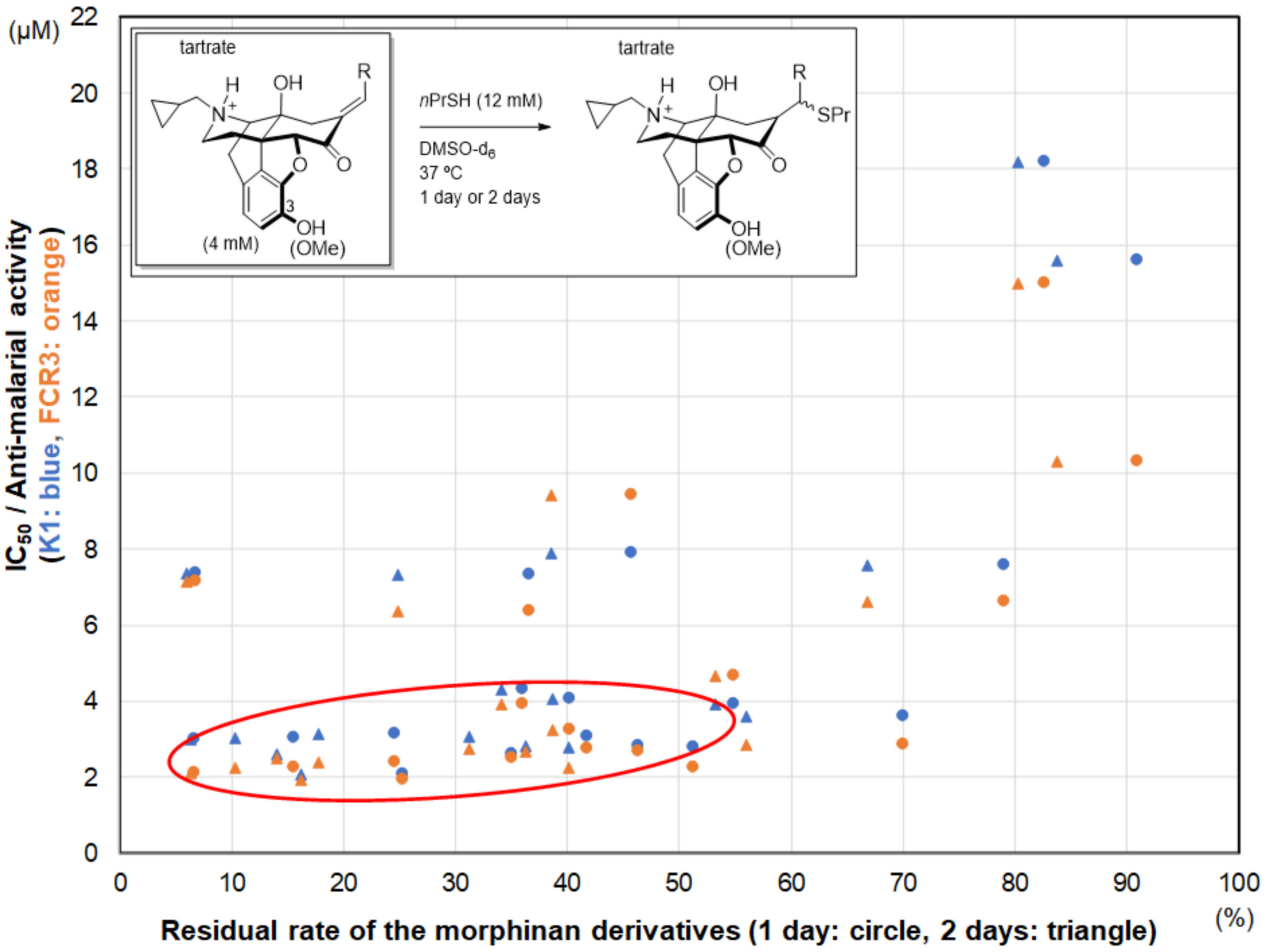
Relationship between antimalarial activities of the BNTX derivatives and the thiol group-trapping ability. Blue symbols represent K1, the CQ-resistant strain, while the orange symbols represent the CQ-sensitive strain, FCR-3. The filled circles (●) indicate the residual rates at day 1 and the filled triangles (▲) represent the day 2 values. ^a^ The residual rates of **1** and **2** after 2.5 days were adopted.

Furthermore, we created a correlation diagram in which the antimalarial activities (IC_50_ values) of the BNTX derivatives shown in Table 1 were on the vertical axis, and the residual rate (%) of the individual derivatives shown in Table 2 was on the horizontal axis, with the exception of compounds **17** and **20** (Figure 3). The data showed a clear tendency for the morphinan derivatives with higher thiol group-trapping ability to have higher antimalarial activity (shown enclosed in the red circle in Figure 3). In contrast, the correlation diagram also showed that a lower ability to capture thiol groups was associated with a lower antimalarial activity of the derivatives. These experimental results partly support the recent research reports on thiol group-trapping effects by other laboratories [24,25,26,27,28,29]. Thus, as we initially described our hypothesis, the antiprotozoal activity (antimalarial or antitrichomonal activity) of the BNTX (**1**) and its derivatives with a Michael acceptor may be expressed by inhibiting the respective antioxidant system of infectious protozoa. In the near future, we aim to further elucidate the mechanism of antimalarial activity of the morphinan compounds by applying these results to in vivo assays, which we have performed in previous studies. When evaluating antimalarial activity of the morphinan derivatives, the examination of the thiol group-trapping ability through an organic chemistry approach might become a reliable primary screening tool.

## 3. Materials and Methods 

All melting points were determined on a Yanaco MP-500P melting point (Mp) apparatus (Tokyo, Japan) and were uncorrected. Infrared spectra (IR) were recorded with a JASCO FT/IR 4100 spectrophotometer (Tokyo, Japan). ^1^H and ^13^C NMR spectra data were obtained with JEOL JNM-ECS 400 instruments (Tokyo, Japan). Chemical shifts are quoted in ppm using tetramethylsilane (δ = 0 ppm) as the reference for ^1^H NMR spectroscopy (JEOL RESONANCE, Tokyo, Japan), CDCl_3_ (δ = 77.0 ppm) for ^13^C NMR spectroscopy. Mass spectra were measured with a JEOL JMS-T100LP spectrometer. Elemental analysis was performed with a J-SCIENCE MICRO CORDER JM-10 model analyzer. Column chromatography was carried out on silica gel (Fuji Silysia, CHROMATOREX PSQ60B, Aichi, Japan). Thin layer chromatography (TLC) and preparative TLC were performed on Merck Kieselgel 60 F_254_ (0.25 mm and 0.50 mm) plates (Darmstadt, Germany). All reactions were performed under an argon atmosphere. Synthetic procedures and analytical data for the compounds **1**–**7**, **16**, **17**, **19**, and **20** were reported in our previous paper [11]. Additionally, the analytical data of compound 8 have been reported [30].

### 3.1. Experimental Data

(12bS)-6-((E)-3-Bromo-5-nitrobenzylidene)-3-(cyclopropylmethyl)-4a,9-dihydroxy-2,3,4,4a,5,6-hexahydro-1H-4,12-methanobenzofuro[3,2-e]isoquinolin-7(7aH)-one (**9**): IR (neat) cm^−1^: 3348, 2925, 2831, 1695, 1533, 1347, 1051. ^1^H NMR (400 MHz, CDCl_3_): δ (ppm) 0.12−0.17 (m, 2H), 0.54−0.58 (m, 2H), 0.83−0.89 (m, 1H), 1.67 (d, J = 13.3 Hz, 1H), 2.30−2.43 (m, 5H), 2.66−2.76 (m, 2H), 2.85 (d, J = 15.1 Hz, 1H), 3.13−3.24 (m, 2H), 4.71 (s, 1H), 6.66 (d, J = 8.2 Hz, 1H), 6.76 (d, J = 8.2 Hz, 1H), 7.46 (d, J = 2.3 Hz, 1H), 7.79 (dd, J = 1.8, 1.8 Hz, 1H), 8.15 (dd, J = 1.8, 1.8 Hz, 1H), 8.31 (dd, J = 1.8, 1.8 Hz, 1H). The OH peaks were not observed. ^13^C NMR (100 MHz, CDCl_3_): δ (ppm) 3.7, 4.2, 9.2, 22.8, 31.6, 33.5, 43.3, 48.1, 59.3, 61.6, 70.6, 89.9, 117.8, 120.4, 123.0 (× 2), 124.4, 126.3, 129.4, 135.1, 136.5, 138.3, 138.4 (× 2), 143.7, 148.7, 198.7. HR-MS (ESI): m/z [M + H]^+^ calcd for C_27_H_26_BrN_2_O_6_: 553.09742, found: 553.09566.

**9**∙*tartrate*: Mp (dec) 166.0−168.0 °C. Anal. Calcd for C_27_H_25_BrN_2_O_6_∙C_4_H_6_O_6_∙1.9H_2_O: C, 50.47; H, 4.75; N, 3.80, found: C, 50.34; H, 4.91; N, 3.65.

(4R,4aS,7aR,12bS,E)-3-(Cyclopropylmethyl)-4a,9-dihydroxy-6-((perfluorophenyl)methylene)-2,3,4,4a,5,6-hexahydro-1H-4,12-methanobenzofuro[3,2-e]isoquinolin-7(7aH)-one (**10**): IR (KBr) cm^−1^: 3377, 2927, 1698, 1521, 1496. ^1^H NMR (400 MHz, CDCl_3_): δ (ppm) 0.11−0.15 (m, 2H), 0.52−0.55 (m, 2H), 0.81−0.84 (m, 1H), 1.63−1.66 (m, 1H), 2.24−2.50 (m, 6H), 2.64−2.74 (m, 2H), 3.13 (d, J = 18.8 Hz, 1H), 3.19 (d, J = 6.0 Hz, 1H), 4.72 (s, 1H), 6.66 (d, J = 8.2 Hz, 1H), 6.75 (d, J = 8.2 Hz, 1H), 7.21 (s, 1H). The OH peaks were not observed. ^13^C NMR (100 MHz, CDCl_3_): δ (ppm) 3.6, 4.2, 9.2, 22.7, 31.6, 35.1, 43.4, 48.1, 59.3, 61.4, 70.2, 89.7, 117.8, 120.4, 123.5, 124.4, 129.3, 138.3, 139.7, 143.5, 197.9. Some aromatic carbons were not observed. HR-MS (ESI): m/z [M + H]^+^ calcd for C_27_H_23_F_5_NO_4_: 520.15472, found: 520.15331.

**10**∙*tartrate*: Mp (dec) 178.2−179.9 °C. Anal. Calcd for C_27_H_22_F_5_NO_4_∙C_4_H_6_O_6_∙2H_2_O: C, 52.77; H, 4.57; N, 1.99, found: C, 52.82; H, 4.68; N, 2.23.

(12bS)-3-(Cyclopropylmethyl)-4a,9-dihydroxy-6-((E)-4-isothiocyanatobenzylidene)-2,3,4,4a,5,6-hexahydro-1H-4,12-methanobenzofuro[3,2-e]isoquinolin-7(7aH)-one (**11**): IR (KBr) cm^−1^: 3409, 2924, 2096, 1685, 1596.

^1^H NMR (400 MHz, CDCl_3_): δ (ppm) 0.13−0.15 (m, 2H), 0.54−0.56 (m, 2H), 0.83−0.88 (m, 1H), 1.66−1.68 (m, 1H), 2.26−2.48 (m, 5H), 2.64−2.74 (m, 2H), 2.92 (d, *J* = 15.6 Hz, 1H), 3.15 (d, *J* = 18.8 Hz, 1H), 3.21 (d, *J* = 6.0 Hz, 1H), 4.69 (s, 1H), 6.65 (d, *J* = 8.0 Hz, 1H), 6.75 (d, *J* = 8.0 Hz, 1H), 7.21 (d, *J* = 8.2 Hz, 2H), 7.34 (d, *J* = 8.2 Hz, 2H), 7.58 (d, *J* = 8.3 Hz, 1H). The OH peaks were not observed. ^13^C NMR (100 MHz, CDCl_3_): δ (ppm) 3.7, 4.1, 9.3, 22.8, 31.7, 33.7, 43.3, 47.8, 59.4, 61.6, 70.2, 89.9, 117.7, 120.2, 124.5, 125.8 (× 2), 129.7, 131.4 (× 2), 131.5, 133.3, 134.1, 136.4, 138.3, 138.6, 143.7, 198.6. HR-MS (ESI): *m*/*z* [M + H]^+^ calcd for C_28_H_27_N_2_O_4_S: 487.16915, found: 487.16863.

**11**∙*hydrochloride*: Mp (dec) 189.6−191.3 °C. Anal. Calcd for C_28_H_26_N_2_O_4_S∙HCl∙2.3H_2_O: C, 59.58; H, 5.64; N, 4.96, found: C, 59.42; H, 5.55; N, 4.82.

N-(4-((E)-((4R,4aS,7aR,12bS)-3-(Cyclopropylmethyl)-4a,9-dihydroxy-7-oxo-1,2,3,4,4a,5,7,7a-octahydro-6H-4,12-methanobenzofuro[3,2-e]isoquinolin-6-ylidene)methyl)phenyl)acrylamide (**12**): IR (KBr) cm^−1^: 3435, 2925, 1672, 1592. ^1^H NMR (400 MHz, CDCl_3_): δ (ppm) 0.13−0.16 (m, 2H), 0.52−0.57 (m, 2H), 0.83−0.86 (m, 1H), 1.64−1.66 (m, 1H), 2.26−2.47 (m, 5H), 2.66−2.72 (m, 2H), 3.00 (d, J = 15.6 Hz, 1H), 3.15 (d, J = 18.8 Hz, 1H), 3.24 (d, J = 6.4 Hz, 1H), 4.68 (s, 1H), 5.78 (dd, J = 10.1, 1.1 Hz, 1H), 6.26 (dd, J = 16.7, 10.7 Hz, 1H), 6.44 (dd, J = 16.7, 1.1 Hz, 1H), 6.64 (d, J = 8.2 Hz, 1H), 6.74 (d, J = 8.2 Hz, 1H), 7.28 (d, J = 8.2 Hz, 2H), 7.57−7.63 (m, 4H). The OH peaks were not observed. ^13^C NMR (100 MHz, CDCl_3_): δ (ppm) 3.6, 4.2, 9.3, 22.9, 31.8, 33.8, 43.4, 47.7, 59.4, 61.5, 70.2, 90.0, 117.7, 119.5 (× 2), 120.1, 124.6, 128.4, 129.8, 130.9, 131.1, 131.4 (× 2), 131.5, 138.4, 138.5, 140.1, 143.8, 163.6, 198.4. HR-MS (ESI): m/z [M + H]^+^ calcd for C_30_H_31_N_2_O_5_: 499.22330, found: 499.22469.

**12**∙*tartrate*: Mp (dec) 202.0−203.9 °C. Anal. Calcd for C_30_H_30_N_2_O_5_∙C_4_H_6_O_6_∙2.8H_2_O: C, 58.41; H, 6.00; N, 4.01, found: C, 58.46; H, 5.81; N, 4.11.

(4R,4aS,7aR,12bS,E)-3-(Cyclopropylmethyl)-6-((6,6-dimethoxy-3-oxocyclohexa-1,4-dien-1-yl)methylene)-4a,9-dihydroxy-2,3,4,4a,5,6-hexahydro-1H-4,12-methanobenzofuro[3,2-e]isoquinolin-7(7aH)-one (**13**): IR (KBr) cm^−1^: 3417, 2937, 1671, 1632. ^1^H NMR (400 MHz, CDCl_3_): δ (ppm) 0.14−0.15 (m, 2H), 0.54−0.59 (m, 2H), 0.83−0.86 (m, 1H), 1.63−1.66 (m, 1H), 2.23−2.40 (m, 4H), 2.46 (dd, J = 12.0, 6.2 Hz, 1H), 2.65 (dd, J = 18.5, 6.4 Hz, 1H), 2.73 (dd, J = 12.0, 4.1 Hz, 1H), 2.93 (d, J = 15.1 Hz, 1H), 3.13 (d, J = 18.5 Hz, 1H), 3.21 (d, J = 6.4 Hz, 1H), 3.25 (s, 6H), 4.69 (s, 1H), 6.29 (s, 1H), 6.44 (dd, J = 10.4, 2.1 Hz, 1H), 6.64 (d, J = 8.2 Hz, 1H), 6.74 (d, J = 8.2 Hz, 1H), 6.81 (d, J = 10.4 Hz, 1H), 7.27 (s, 1H). The OH peaks were not observed. ^13^C NMR (100 MHz, CDCl_3_): δ (ppm) 3.7, 4.1, 9.3, 22.7, 31.5, 34.6, 43.3, 48.0, 51.2, 51.3, 59.4, 61.6, 70.4, 89.9, 94.9, 117.6, 120.2, 124.6, 129.5, 131.1, 131.7, 132.9, 138.2, 138.4, 143.6, 144.4, 151.4, 185.0, 198.5. HR-MS (ESI): m/z [M + H]^+^ calcd for C_29_H_32_NO_7_: 506.21788, found: 506.21626.

**13**∙*tartrate*: Mp (dec) 218.6−220.5 °C. Anal. Calcd for C_29_H_31_NO_7_∙C_4_H_6_O_6_∙3.5H_2_O: C, 55.15; H, 6.17; N, 1.95, found: C, 55.17; H, 5.79; N, 1.92.

(4R,4aS,7aR,12bS,E)-6-(Cyclohexylmethylene)-3-(cyclopropylmethyl)-4a,9-dihydroxy-2,3,4,4a,5,6-hexahydro-1H-4,12-methanobenzofuro[3,2-e]isoquinolin-7(7aH)-one (**14**): IR (KBr) cm^−1^: 3422, 2925, 1686. ^1^H NMR (400 MHz, CDCl_3_): δ (ppm) 0.14−0.15 (m, 2H), 0.52−0.60 (m, 2H), 0.86−0.87 (m, 1H), 1.06−1.29 (m, 5H), 1.44 (d, J = 12.8 Hz, 1H), 1.59−1.73 (m, 5H), 2.09 (dd, J = 15.1, 2.8 Hz, 1H), 2.21−2.46 (m, 5H), 2.60−2.73 (m, 3H), 3.10 (d, J = 18.3 Hz, 1H), 3.24 (s, 1H), 4.59 (s, 1H), 6.55−6.60 (m, 2H), 6.70 (d, J = 8.2 Hz, 1H). The OH peaks were not observed. ^13^C NMR (100 MHz, CDCl_3_): δ (ppm) 3.8, 4.1, 9.3, 22.9, 25.4, 25.5, 25.7, 31.4 (× 2), 31.9, 32.5, 37.2, 43.4, 47.9, 59.4, 61.8, 70.1, 90.1, 117.5, 119.9, 124.4, 129.8, 130.5, 138.3, 143.6, 149.4, 198.7. HR-MS (ESI): m/z [M + H]^+^ calcd for C_27_H_34_NO_4_: 436.24878, found: 436.24706. **14**∙tartrate: Mp (dec) 176.8−178.8 °C. Anal. Calcd for C_27_H_33_NO_4_∙C_4_H_6_O_6_∙1.8H_2_O: C, 60.24; H, 6.95; N, 2.27, found: C, 60.39; H, 6.92; N, 2.14.

(4R,4aS,7aR,12bS,E)-6-(Cyclobutylmethylene)-3-(cyclopropylmethyl)-4a,9-dihydroxy-2,3,4,4a,5,6-hexahydro-1H-4,12-methanobenzofuro[3,2-e]isoquinolin-7(7aH)-one (**15**): IR (KBr) cm^−1^: 3419, 2936, 1685, 1612. ^1^H NMR (400 MHz, CDCl_3_): δ (ppm) 0.15−0.16 (m, 2H), 0.56−0.57 (m, 2H), 0.86−0.89 (m, 1H), 1.60−1.63 (m, 1H), 1.85−2.12 (m, 6H), 2.21−2.39 (m, 4H), 2.46 (dd, J = 12.6, 6.2 Hz, 1H), 2.56−2.65 (m, 2H), 2.70−2.74 (m, 1H), 3.09−3.24 (m, 3H), 4.60 (s, 1H), 6.60 (d, J = 8.2 Hz, 1H), 6.71 (d, J = 8.2 Hz, 1H), 6.88 (dd, J = 8.7, 2.3 Hz, 1H). The OH peaks were not observed. ^13^C NMR (100 MHz, CDCl_3_): δ (ppm) 3.7, 4.1, 9.3, 19.1, 22.8, 28.65, 28.67, 31.7, 32.5, 34.3, 43.3, 47.9, 59.4, 61.7, 70.0, 90.1, 117.3, 119.9, 124.6, 129.9, 130.3, 138.2, 143.6, 148.9, 197.9. HR-MS (ESI): m/z [M + H]^+^ calcd for C_25_H_30_NO_4_: 408.21748, found: 408.21657. 

**15**∙*tartrate*: Mp (dec) 166.9−168.9 °C. Anal. Calcd for C_25_H_29_NO_4_∙C_4_H_6_O_6_∙1.4H_2_O: C, 59.76; H, 6.54; N, 2.40, found: C, 59.73; H, 6.72; N, 2.48.

(4R,4aS,7aR,12bS,E)-3-(Cyclopropylmethyl)-6-(cyclopropylmethylene)-4a,9-dihydroxy-2,3,4,4a,5,6-hexahydro-1H-4,12-methanobenzofuro[3,2-e]isoquinolin-7(7aH)-one (**18**): Mp (dec) 222.0−223.8 °C. IR (KBr) cm^−1^: 3399, 2924, 2821, 1678, 1328. ^1^H NMR (400 MHz, CDCl_3_): δ (ppm) 0.14−0.18 (m, 2H), 0.54−0.59 (m, 2H), 0.63−0.75 (m, 2H), 0.87−1.01 (m, 3H), 1.51−1.55 (m, 1H), 1.62−1.65 (m, 1H), 2.23−2.50 (m, 5H), 2.61−2.73 (m, 2H), 2.80 (d, J = 15.6 Hz, 1H), 3.14 (d, J = 18.8 Hz, 1H), 3.27 (d, J = 6.0 Hz, 1H), 4.61 (s, 1H), 5.28 (brs, 1H), 6.21 (dd, J = 11.2, 2.1 Hz, 1H), 6.60 (d, J = 8.2 Hz, 1H), 6.71 (d, J = 8.2 Hz, 1H). The OH peak was not observed. ^13^C NMR (100 MHz, CDCl_3_): δ (ppm) 3.6, 4.1, 9.35, 9.38, 9.8, 12.2, 22.9, 31.8, 32.4, 43.4, 47.7, 59.4, 61.6, 69.9, 90.0, 117.3, 119.8, 124.6, 129.3, 130.0, 138.2, 143.7, 150.9, 196.1. HR-MS (ESI): m/z [M + H]^+^ calcd for C_24_H_28_NO_4_: 394.20183, found: 394.20102.

**18**∙*tartrate*: Mp (dec) 173.0−175.0 °C. Anal. Calcd for C_24_H_27_NO_4_∙C_4_H_6_O_6_∙1.2H_2_O: C, 59.50; H, 6.31; N, 2.48, found: C, 59.67; H, 6.33; N, 2.22.

### 3.2. Antimalarial Assay

The evaluation of antimalarial activity was conducted as previously reported [21]. Briefly, cultured *P. falciparum* (chloroquine sensitive FCR3 strain and chloroquine resistant K1 strain) in Type A^+^ blood was seeded in 96-well culture plates (parasitemia 0.5%–1%, hematocrit 2.0%) and incubated with test drugs for 72 h in RPMI medium supplemented with 10% human plasma at 37 ⁰C, under 93% N_2_, 4% CO_2_, and 3% O_2_. After incubation, parasite lactate dehydrogenase activity was assayed to determine parasite growth and calculate the antimalarial activity in comparison with the controls that had received no drugs. This study was approved by “Kitasato Institute Hospital Research Ethics Committee (No12102)” on donated human blood from volunteers.

## 4. Conclusions

In conclusion, the antimalarial activities of the BNTX derivatives with a Michael acceptor were evaluated using CQ-resistant K1 and -sensitive FCR3 strains. Moreover, the thiol group-trapping abilities of the derivatives were also investigated relatively, using ^1^H-NMR experiments. The experimental results strongly supported a correlation between the antimalarial activity and the chemical reactivity of the BNTX derivatives with 1-propanethiol. Thus, the measurement of the thiol group-trapping ability of the derivatives with a Michael acceptor could become a reliable and valid screening technique for antimalarial or related activities.

## Figures and Tables

**Table 1:**
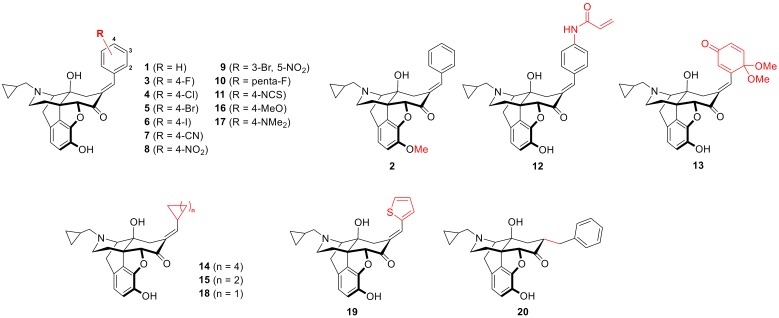
In vitro antimalarial activity of the morphinan derivatives **1**–**20**.

Compound	IC_50_ (μM)		Compound	IC_50_ (μM)	
	K1 ^a^	FCR3 ^b^		K1^a^	FCR3 ^b^
Artemisinin	0.03	0.04	**10** ^d^	7.33	6.38
Chloroquine	0.57	0.07	**11** ^c^	2.60	2.50
BNTX (**1**) ^c^	4.08	3.26	**12** ^d^	3.93	4.66
**2** ^d^	4.31	3.94	**13** ^d^	7.89	9.43
**3** ^d^	3.60	2.86	**14** ^d^	2.08	1.95
**4** ^d^	2.82	2.67	**15** ^d^	7.37	7.16
**5** ^d^	3.07	2.76	**16** ^d^	7.58	6.61
**6** ^d^	2.78	2.24	**17** ^d^	>20.1	N/A
**7** ^d^	3.16	2.38	**18** ^d^	18.3	15.0
**8** ^d^	3.04	2.24	**19** ^d^	15.6	10.3
**9** ^d^	2.99	2.13	**20** ^d^	19.8	>21.5

^a^ chloroquine-resistant *P. falciparum* strain. ^b^ chloroquine-sensitive *P. falciparum* strain. ^c^ hydrochloride. ^d^ tartrate.

**Table 2:** The residual rate of the derivatives **1**–**20** after 1 or 2 days.

Compound	Residual Rate (%)		Compound	Residual Rate (%)	
	1 day	2 days		1 day	2 days
**1** ^c^	40.2	38.7 ^b^	**11** ^a^	35.1	14.0
**2** ^c^	36.0	34.1 ^b^	**12** ^c^	54.9	53.2
**3** ^c^	70.0	56.0	**13** ^c^	45.8	38.6
**4** ^c^	46.4	36.3	**14** ^c^	25.3	16.2
**5** ^c^	41.8	31.2	**15** ^c^	6.8	6.0
**6** ^c^	51.3	40.1	**16** ^c^	79.0	66.8
**7** ^c^	24.6	17.7	**17** ^c^	90.0	88.3
**8** ^c^	15.6	10.3	**18** ^c^	82.6	80.2
**9** ^c^	6.7	6.3	**19** ^c^	90.9	83.8
**10** ^c^	36.6	24.8	**20** ^c^	97.9	89.8

^a^ hydrochloride. ^b^ 2.5 days later. ^c^ tartrate.

## Supplementary Materials

The addition reactions of 1-propanethiol (Table 2) in detail are available online.
