# Supplementary material for: Structure-Activity Relationship between Thiol Group-Trapping Ability of Morphinan Compounds with a Michael Acceptor and Anti-Plasmodium falciparum Activities
Source: Molecules. 2020 Mar 2;25(5):1112. doi: 10.3390/molecules25051112 (PMC7179212; doi:10.3390/molecules25051112)
Supplement: Supplementary file 1 [file molecules-25-01112-s001.pdf]

Supporting Information

Structure-activity relationship between thiol group-trapping ability of morphinan compounds with a Michael acceptor and anti-*Plasmodium falciparum* activities

Noriki Kutsumura[a,b], Yasuaki Koyama[b], Tsuyoshi Saitoh[a], Naoshi Yamamoto[a], Yasuyuki Nagumo[a], Yoshiyuki Miyata[c], Rei Hokari[d], Aki Ishiyama[d], Masato Iwatsuki[d], Kazuhiko Otoguro[d], Satoshi Ōmura[d], and Hiroshi Nagase[a,b,*]

[a]*International Institute for Integrative Sleep Medicine (WPI-IIIS), University of Tsukuba, 1-1-1 Tennodai, Tsukuba, Ibaraki 305-8575, Japan*

[b]*Graduate School of Pure and Applied Sciences, University of Tsukuba, 1-1-1 Tennodai, Tsukuba, Ibaraki 305-8571, Japan*

[c]*School of Medicine, Keio University, 35, Shinanomachi, Shinjuku-ku, Tokyo 160-8582, Japan*

[d]*Kitasato Institute for Life Sciences, Kitasato University, 5-9-1 Shirokane, Minato-ku, Tokyo 108-8641, Japan*

E-mail: nagase.hiroshi.gt@u.tsukuba.ac.jp

Table of Contents

1. **Addition reactions of 1-propanethiol …. S2**
2. **^{1}H and ^{13}C NMR spectra …. S6**
3. **Dose-response curves …. S22**

Addition reactions of 1-propanethiol

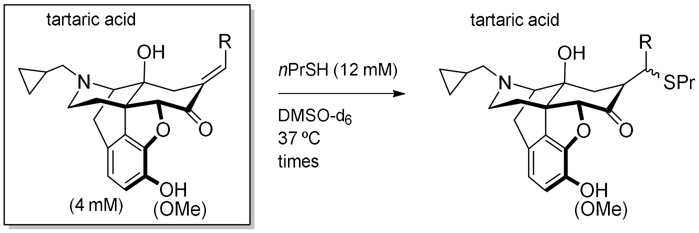

BNTX(**1**)-TA

time (m)	Int. (abn)	ratio (%)
1	1.4635	100
30	1.4482	98.95456
180	1.2421	84.87188
360	0.8775	59.959
720	0.7033	48.05603
1440	0.5877	40.15716
3600	0.5669	38.73591

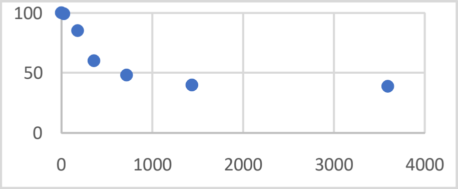

2-TA

time (m)	Int. (abn)	ratio (%)
1	1.4763	100
30	1.3691	92.7386
180	1.2528	84.8608
360	1.083	73.35907
720	0.799	54.12179
1440	0.5316	36.00894
3600	0.503	34.07167

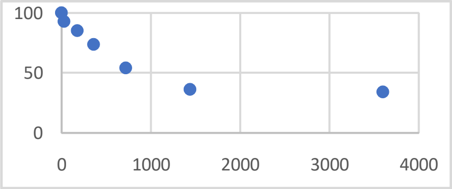

3-TA

time (m)	Int. (abn)	ratio (%)
1	1.5408	100
30	1.496	97.09242
180	1.4494	94.06802
360	1.3888	90.13499
720	1.1489	74.56516
1440	1.0778	69.95067
2880	0.8636	56.04881

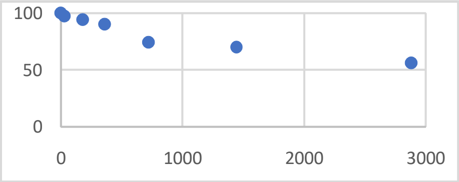

4-TA

time (m)	Int. (abn)	ratio (%)
1	1.7034	100
30	1.6603	97.46977
180	1.2691	74.50393
360	1.0706	62.85077
720	0.9494	55.73559
1440	0.7898	46.36609
2880	0.6175	36.25103

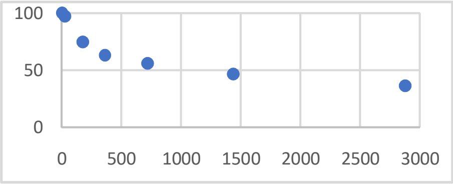

5-TA

time (m)	Int. (abn)	ratio (%)
1	1.6355	100
30	1.5238	93.17028
180	1.4997	91.69673
360	1.0609	64.86701
720	0.8704	53.2192
1440	0.6833	41.77927
2880	0.5097	31.16478

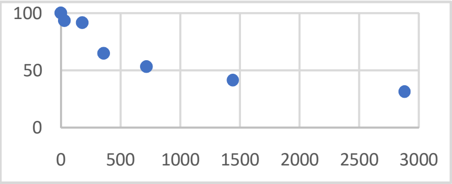

6-TA

time (m)	Int. (abn)	ratio (%)
1	1.7857	100
30	1.747	97.83278
180	1.5714	87.9991
360	1.4898	83.42947
720	1.1893	66.60133
1440	0.9166	51.33001
2880	0.7159	40.09072

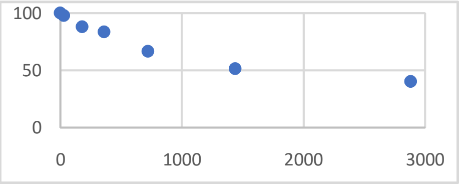

7-TA

time (m)	Int. (abn)	ratio (%)
1	1.6815	100
30	1.5134	90.00297
180	1.1823	70.31222
360	0.9341	55.55159
720	0.6697	39.82753
1440	0.4138	24.60898
2880	0.2968	17.65091

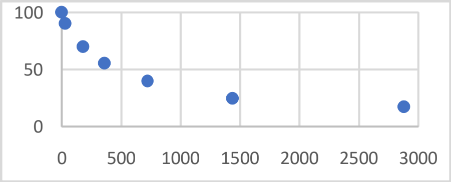

8-TA

time (m)	Int. (abn)	ratio (%)
1	1.4408	100
30	1.2828	89.03387
180	0.8351	57.96086
360	0.5503	38.19406
1440	0.2252	15.63021
2880	0.1478	10.25819

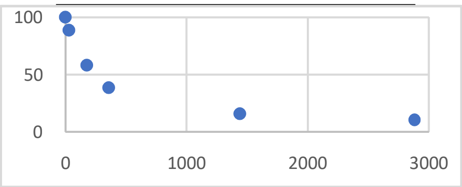

9-TA

time (m)	Int. (abn)	ratio (%)
1	1.3005	100
30	1.0857	83.48328
180	0.4692	36.07843
360	0.1974	15.17878
720	0.109	8.381392
1440	0.0876	6.735871
2880	0.0823	6.328335

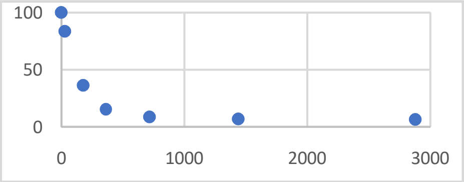

10-TA

time (m)	Int. (abn)	ratio (%)
1	1.0206	100
30	0.9625	94.30727
180	0.846	82.89242
360	0.795	77.89536
1440	0.3733	36.57652
2880	0.2535	24.83833

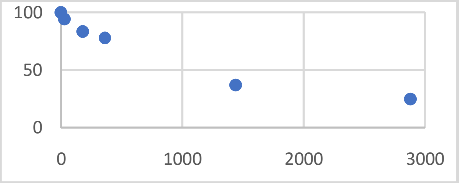

11-HCl

time (m)	Int. (abn)	ratio (%)
1	1.5228	100
30	1.5091	99.10034
180	1.3954	91.63383
360	1.22	80.11558
1440	0.5343	35.08668
2880	0.2135	14.02023

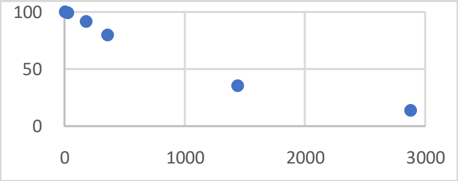

12-TA

time (m)	Int. (abn)	ratio (%)
1	1.4748	100
30	1.3582	92.09384
180	1.3313	90.26987
360	1.3003	88.16789
1440	0.8104	54.94982
2880	0.7852	53.24112

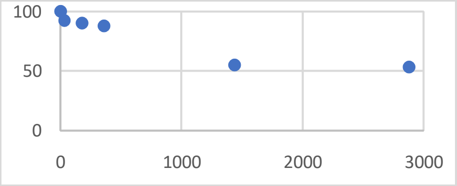

13-TA

time (m)	Int. (abn)	ratio (%)
1	1.1039	100
30	0.9917	89.83604
180	0.8533	77.29867
360	0.8072	73.12257
1440	0.5056	45.80125
2880	0.426	38.59045

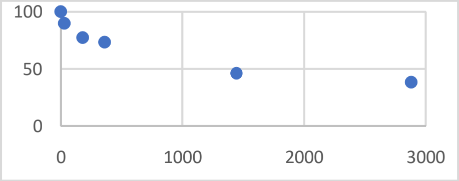

14-TA

time (m)	Int. (abn)	ratio (%)
1	1.8573	100
30	1.752	94.33048
180	1.3312	71.67394
360	1.1317	60.93254
720	0.7609	40.96807
1440	0.4696	25.28401
2880	0.3008	16.19555

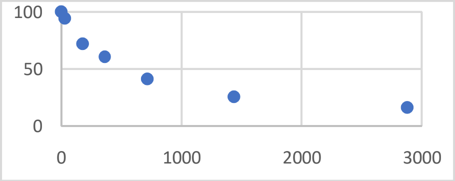

15-TA

time (m)	Int. (abn)	ratio (%)
1	1.101	100
30	0.9527	86.53043
180	0.4669	42.4069
360	0.2204	20.01817
1440	0.0744	6.757493
2880	0.066	5.99455

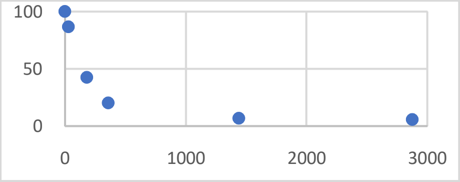

16-TA

time (m)	Int. (abn)	ratio (%)
1	1.7753	100
30	1.7696	99.67893
180	1.5856	89.31448
360	1.5368	86.56565
720	1.4656	82.55506
1440	1.4019	78.96694
2880	1.1861	66.81124

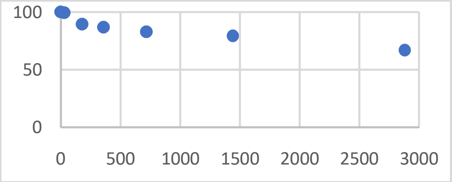

17-TA

time (m)	Int. (abn)	ratio (%)
1	1.7582	100
30	1.7341	98.62928
180	1.6547	94.1133
360	1.6231	92.31601
720	1.6077	91.44011
1440	1.5828	90.02389
2880	1.5522	88.28347

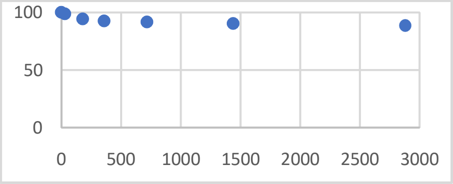

18-TA

time (m)	Int. (abn)	ratio (%)
1	1.561	100
30	1.4408	92.29981
180	1.2508	80.12812
360	1.2896	82.61371
720	1.4004	89.71172
1440	1.2887	82.55605
2880	1.2514	80.16656

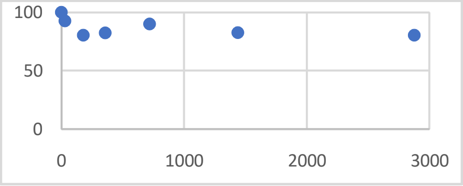

19-TA

time (m)	Int. (abn)	ratio (%)
1	1.8299	100
30	1.8042	98.59555
180	1.8184	99.37155
360	1.5862	86.68233
720	1.4007	76.54517
1440	1.6642	90.94486
2880	1.534	83.82972

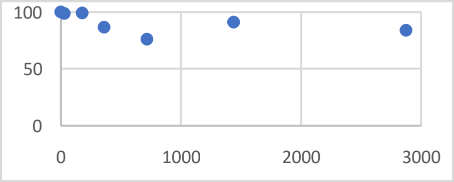

20-TA

time (m)	Int. (abn)	ratio (%)
1	1.7573	100
30	1.7029	96.90434
180	1.5902	90.49109
360	1.6551	94.18426
720	1.491	84.84607
1440	1.7206	97.91157
2880	1.5784	89.81961

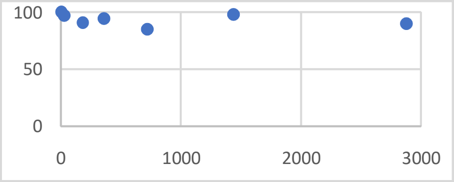

(12bS)-6-((E)-3-Bromo-5-nitrobenzylidene)-3-(cyclopropylmethyl)-4a,9-dihydroxy-2,3,4,4a,5,6-hexahydro-1H-4,12-methanobenzofuro[3,2-e]isoquinolin-7(7aH)-one (**9**)

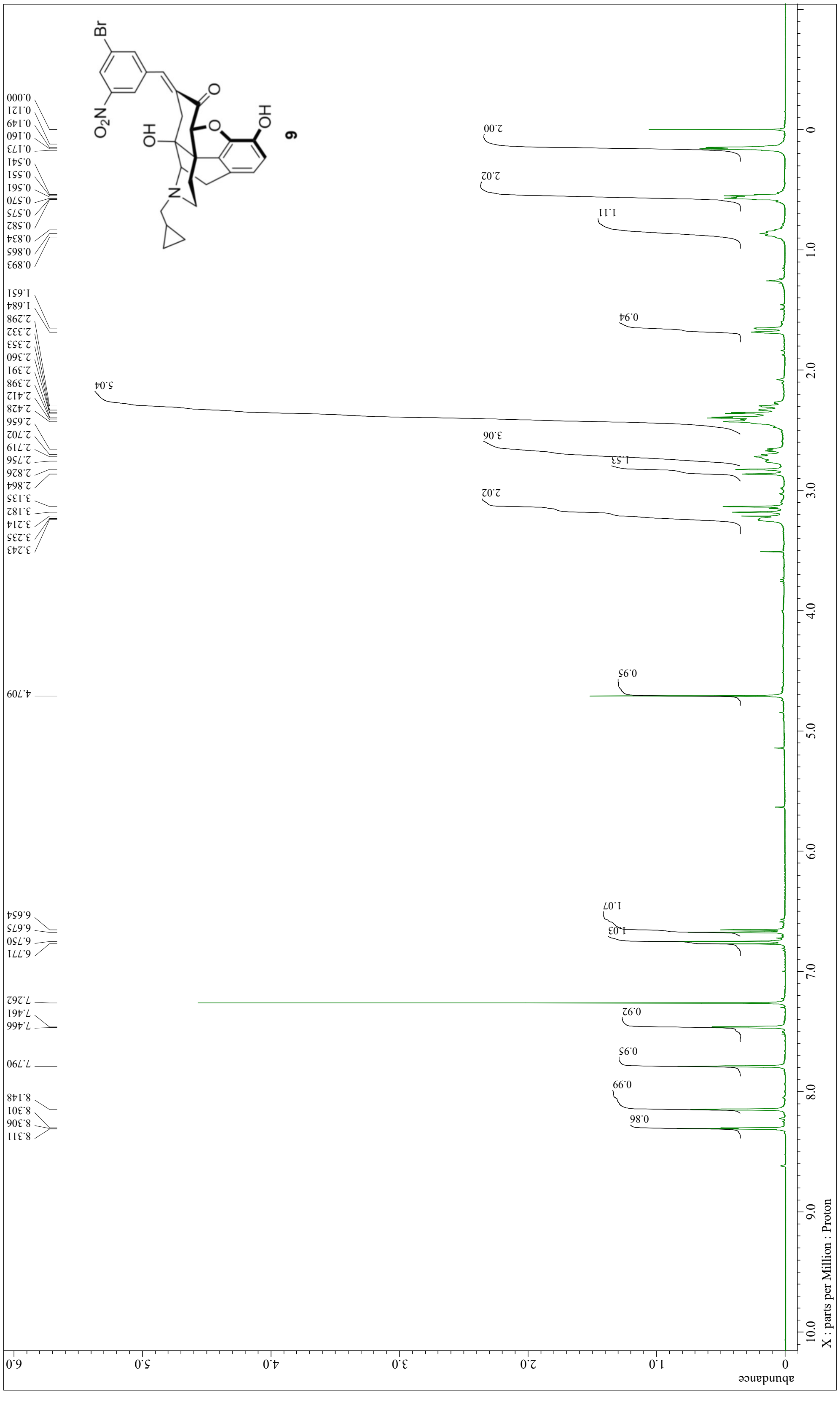

(12bS)-6-((E)-3-Bromo-5-nitrobenzylidene)-3-(cyclopropylmethyl)-4a,9-dihydroxy-2,3,4,4a,5,6-hexahydro-1H-4,12-methanobenzofuro[3,2-e]isoquinolin-7(7aH)-one (**9**)

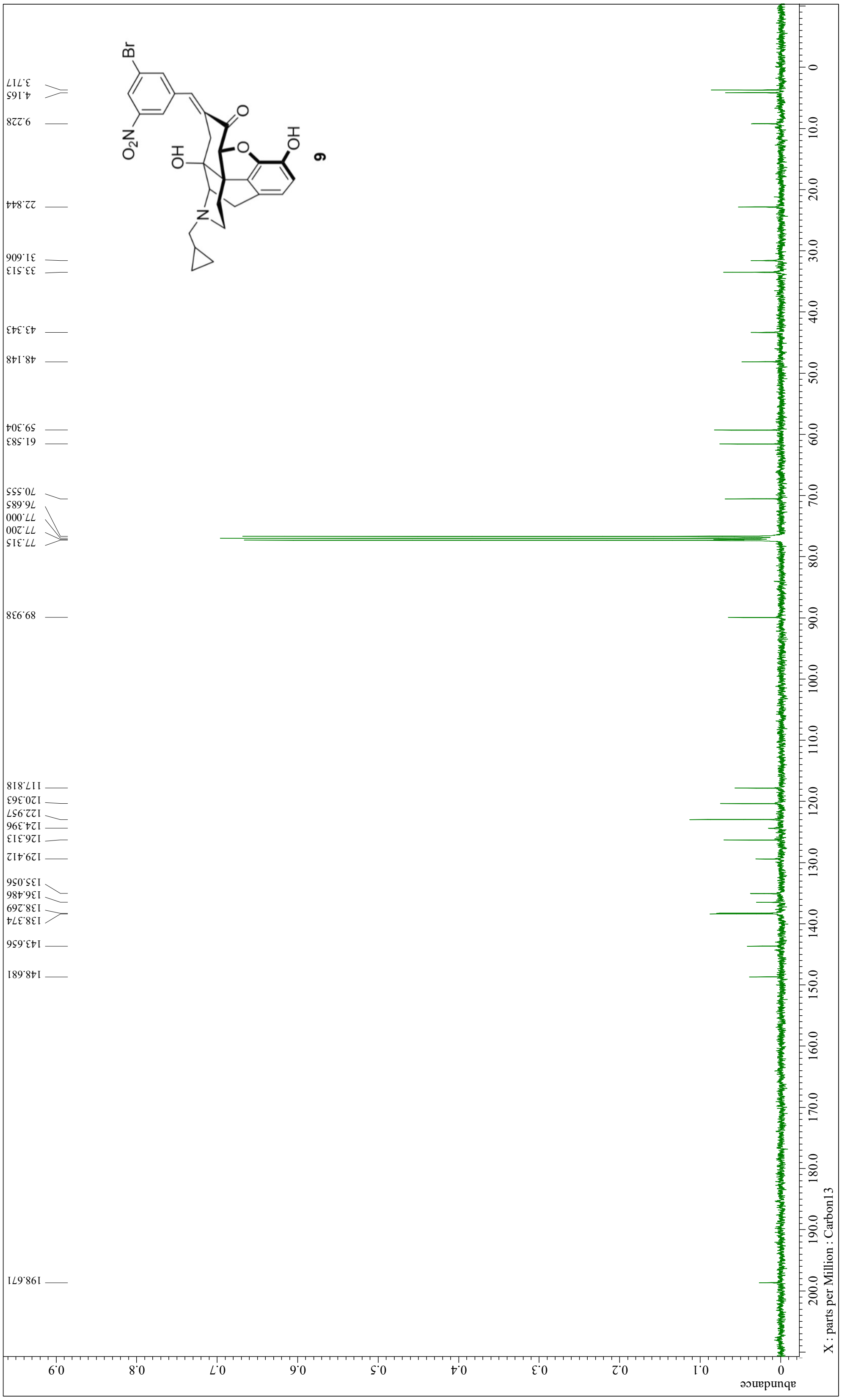

(4R,4aS,7aR,12bS,E)-3-(Cyclopropylmethyl)-4a,9-dihydroxy-6-((perfluorophenyl)methylene)-2,3,4,4a,5,6-hexahydro-1H-4,12-methanobenzofuro[3,2-e]isoquinolin-7(7aH)-one (**10**)

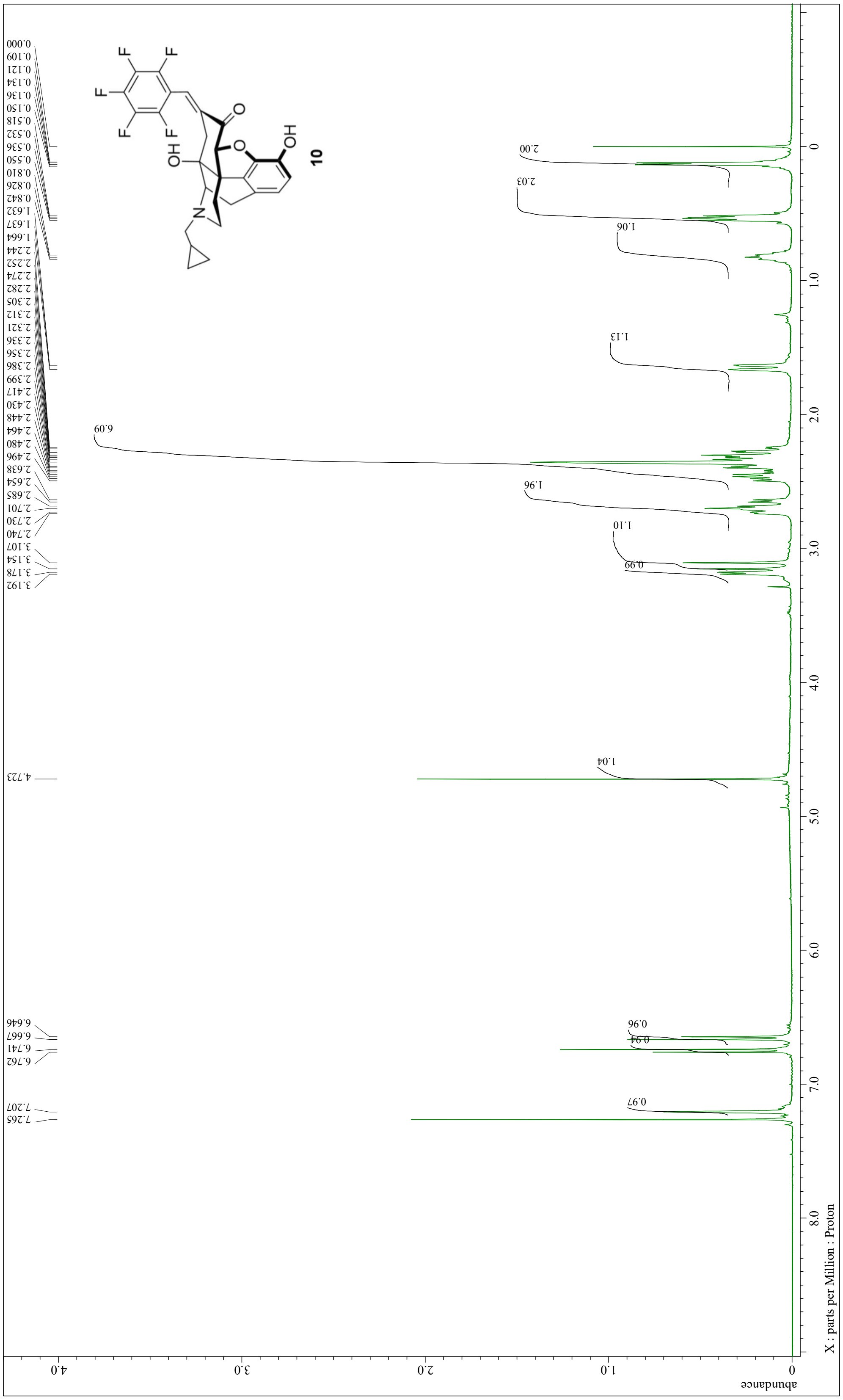

(4R,4aS,7aR,12bS,E)-3-(Cyclopropylmethyl)-4a,9-dihydroxy-6-((perfluorophenyl)methylene)-2,3,4,4a,5,6-hexahydro-1H-4,12-methanobenzofuro[3,2-e]isoquinolin-7(7aH)-one (**10**)

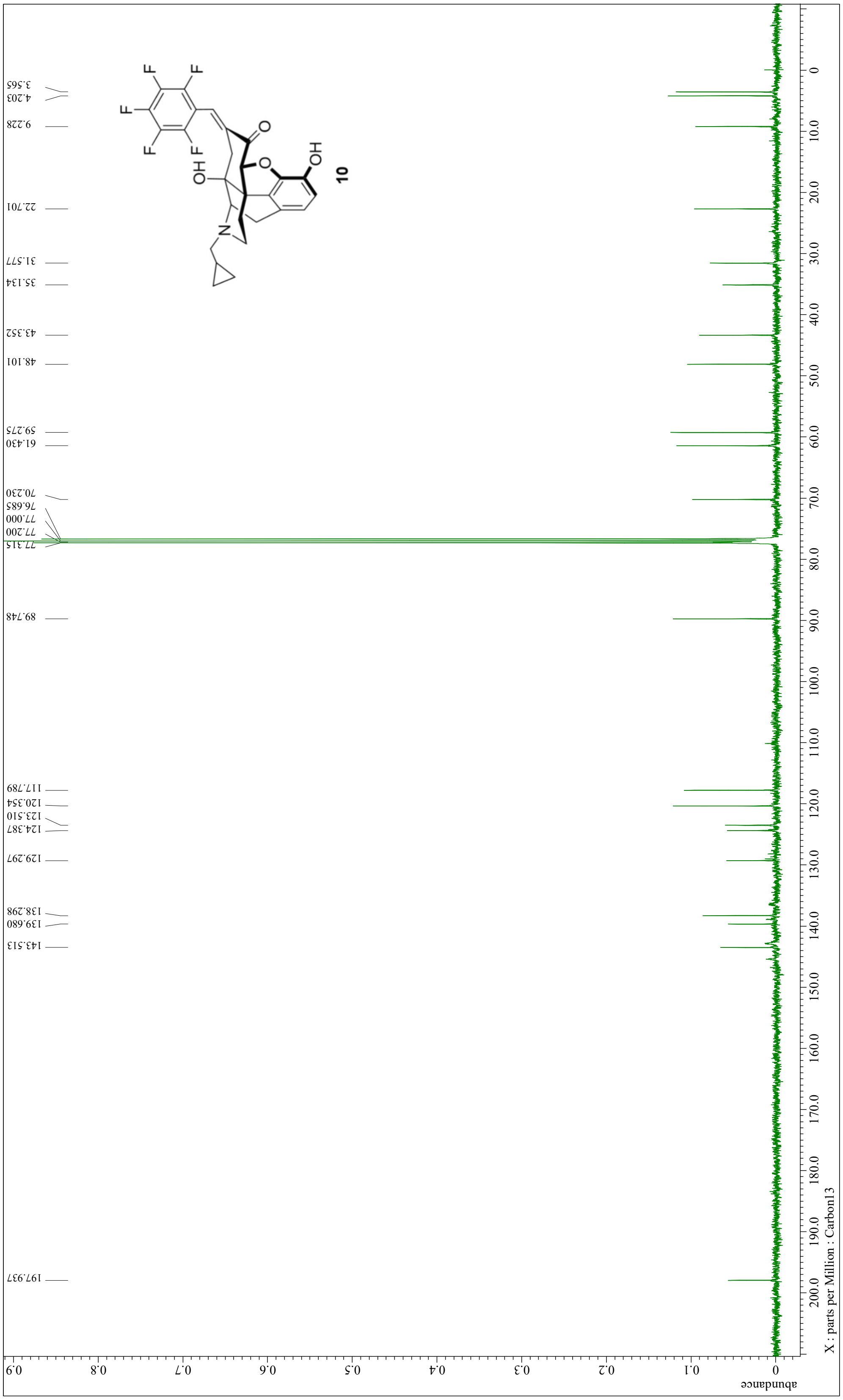

(12bS)-3-(Cyclopropylmethyl)-4a,9-dihydroxy-6-((E)-4-isothiocyanatobenzylidene)-2,3,4,4a,5,6-hexahydro-1H-4,12-methanobenzofuro[3,2-e]isoquinolin-7(7aH)-one (**11**)

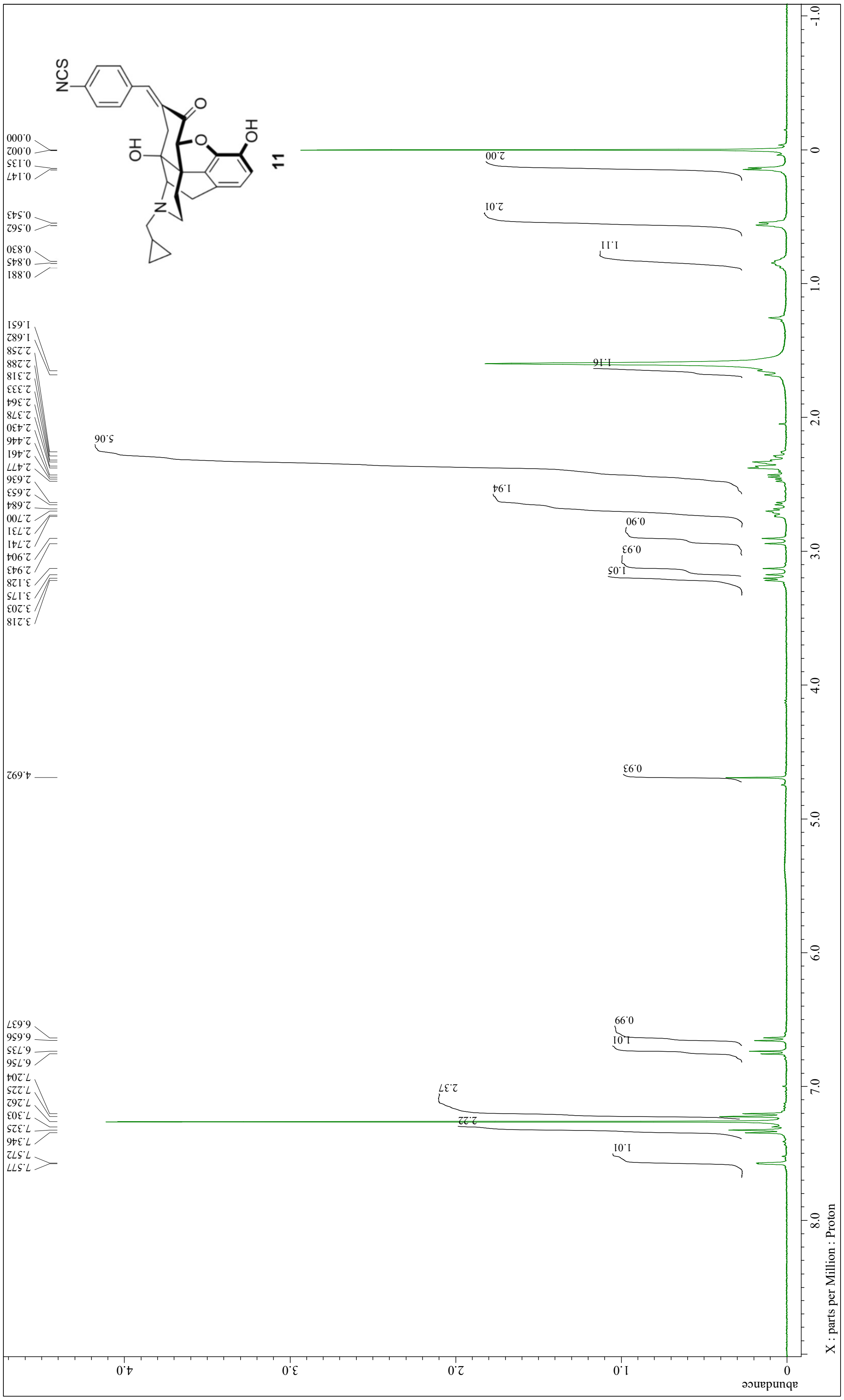

(12bS)-3-(Cyclopropylmethyl)-4a,9-dihydroxy-6-((E)-4-isothiocyanatobenzylidene)-2,3,4,4a,5,6-hexahydro-1H-4,12-methanobenzofuro[3,2-e]isoquinolin-7(7aH)-one (**11**)

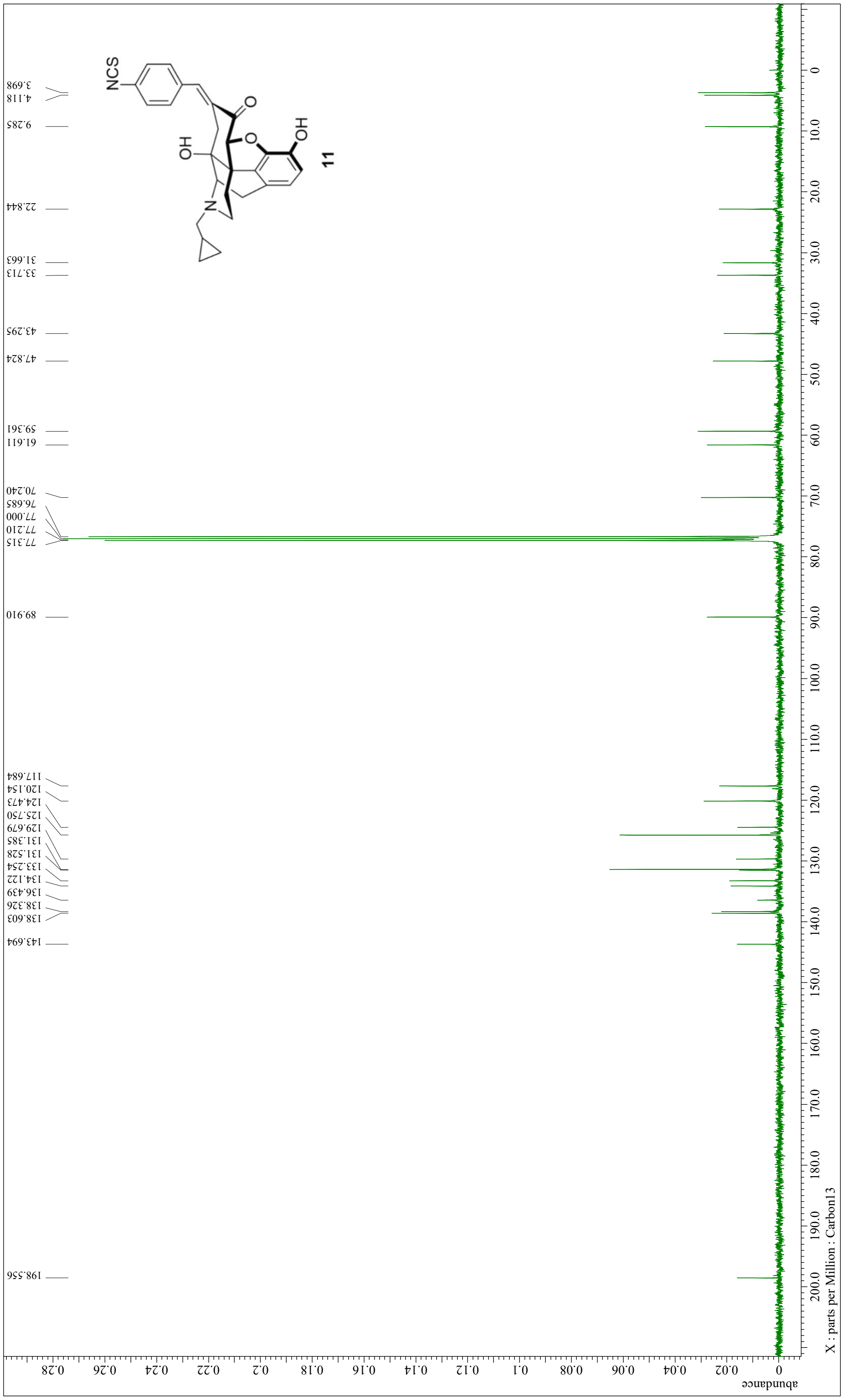

N-(4-((E)-((4R,4aS,7aR,12bS)-3-(Cyclopropylmethyl)-4a,9-dihydroxy-7-oxo-1,2,3,4,4a,5,7,7a-octahydro-6H-4,12-methanobenzofuro[3,2-e]isoquinolin-6-ylidene)methyl)phenyl)acrylamide (**12**)

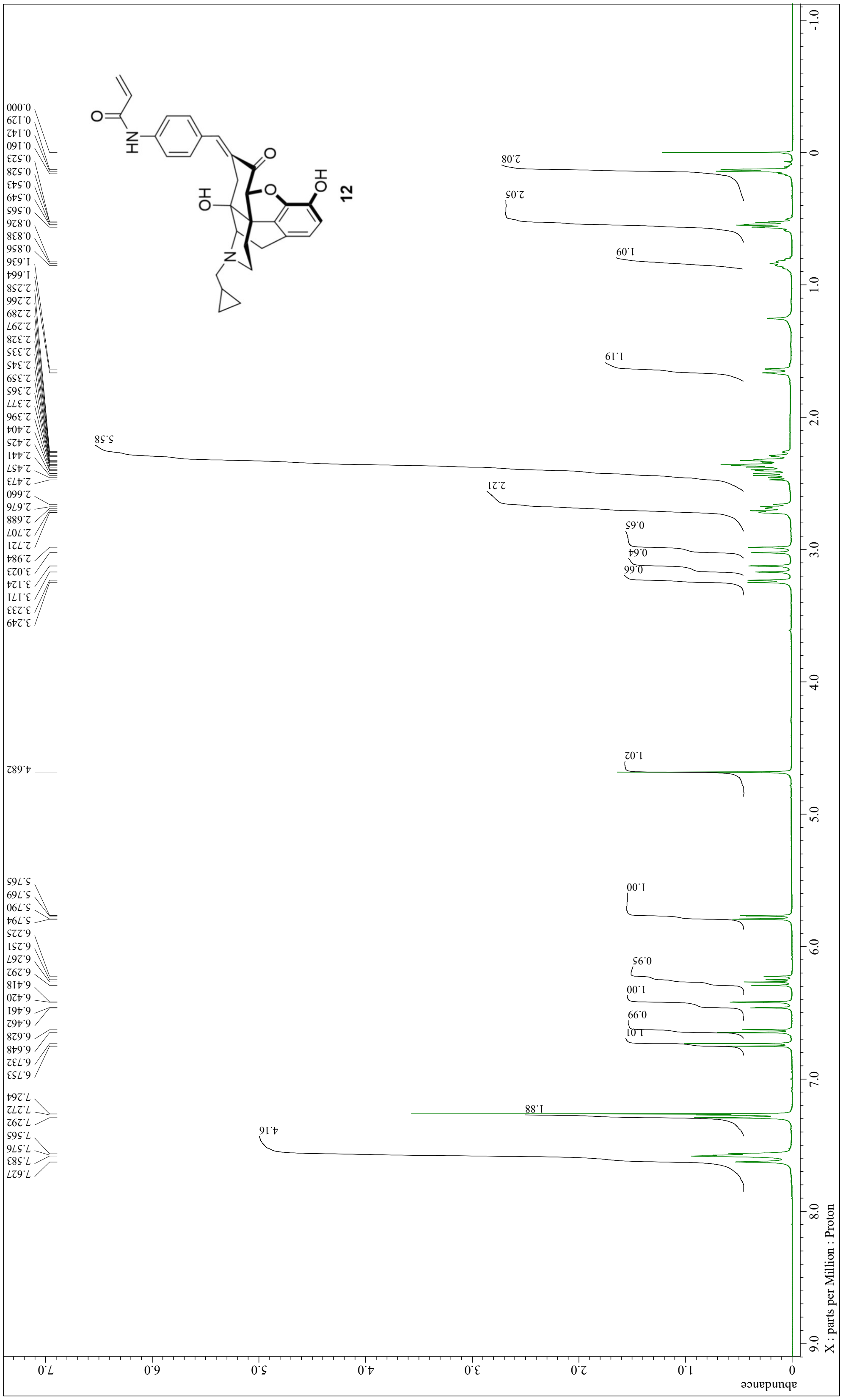

N-(4-((E)-((4R,4aS,7aR,12bS)-3-(Cyclopropylmethyl)-4a,9-dihydroxy-7-oxo-1,2,3,4,4a,5,7,7a-octahydro-6H-4,12-methanobenzofuro[3,2-e]isoquinolin-6-ylidene)methyl)phenyl)acrylamide (**12**)

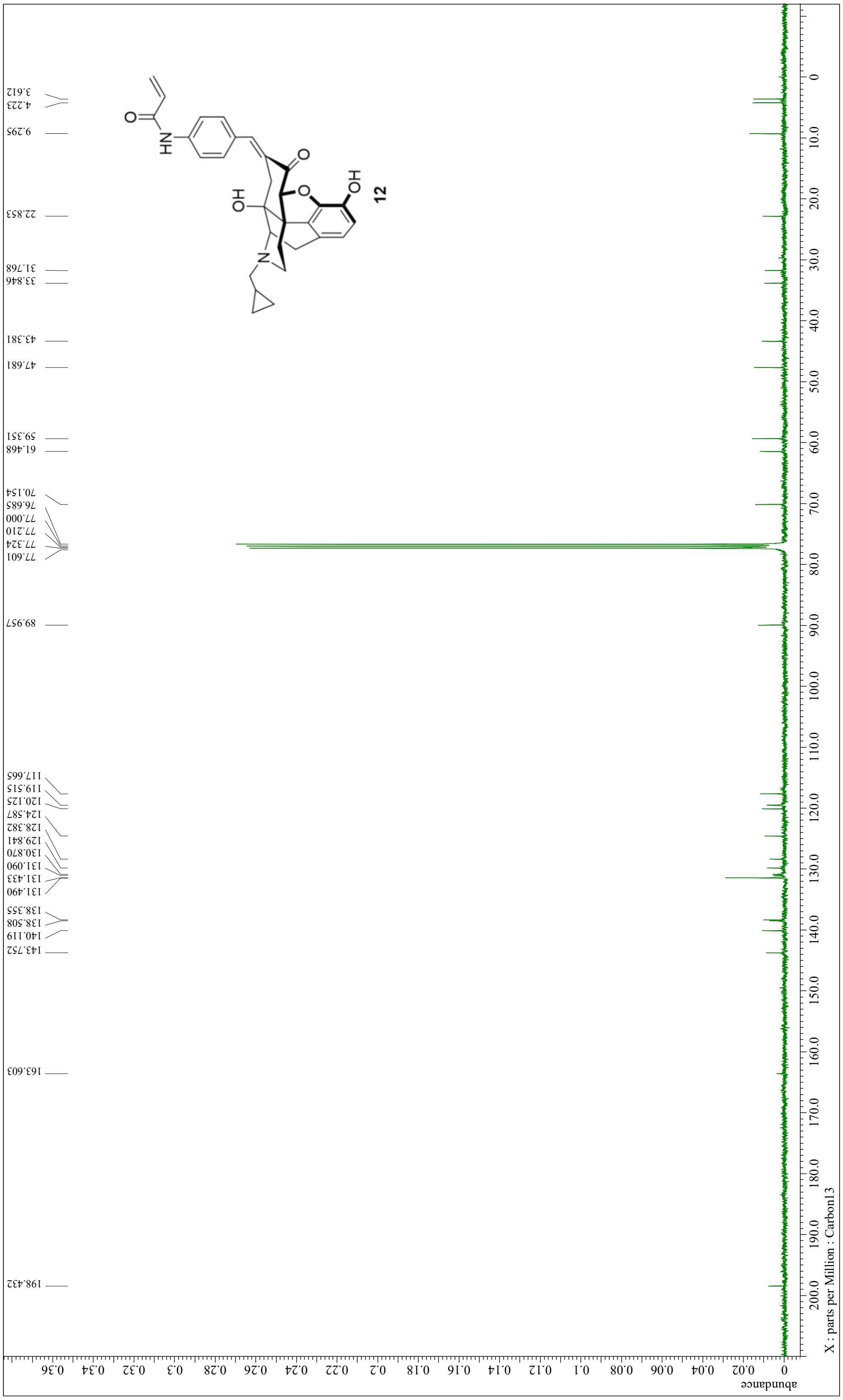

(4R,4aS,7aR,12bS,E)-3-(Cyclopropylmethyl)-6-((6,6-dimethoxy-3-oxocyclohexa-1,4-dien-1-yl)methylene)-4a,9-dihydroxy-2,3,4,4a,5,6-hexahydro-1H-4,12-methanobenzofuro[3,2-e]isoquinolin-7(7aH)-one (**13**)

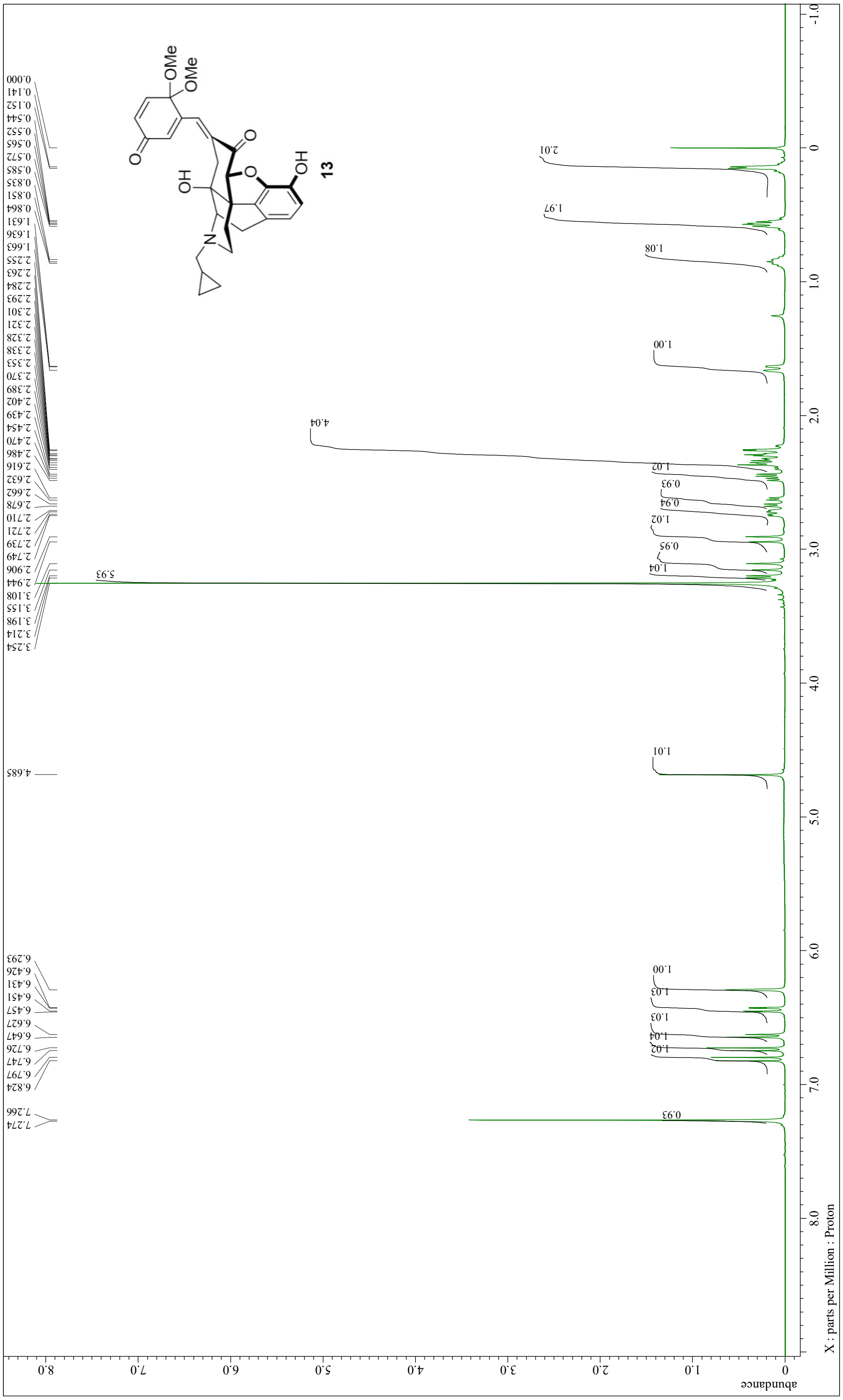

(4R,4aS,7aR,12bS,E)-3-(Cyclopropylmethyl)-6-((6,6-dimethoxy-3-oxocyclohexa-1,4-dien-1-yl)methylene) -4a,9-dihydroxy-2,3,4,4a,5,6-hexahydro-1H-4,12-methanobenzofuro[3,2-e]isoquinolin-7(7aH)-one (**13**)

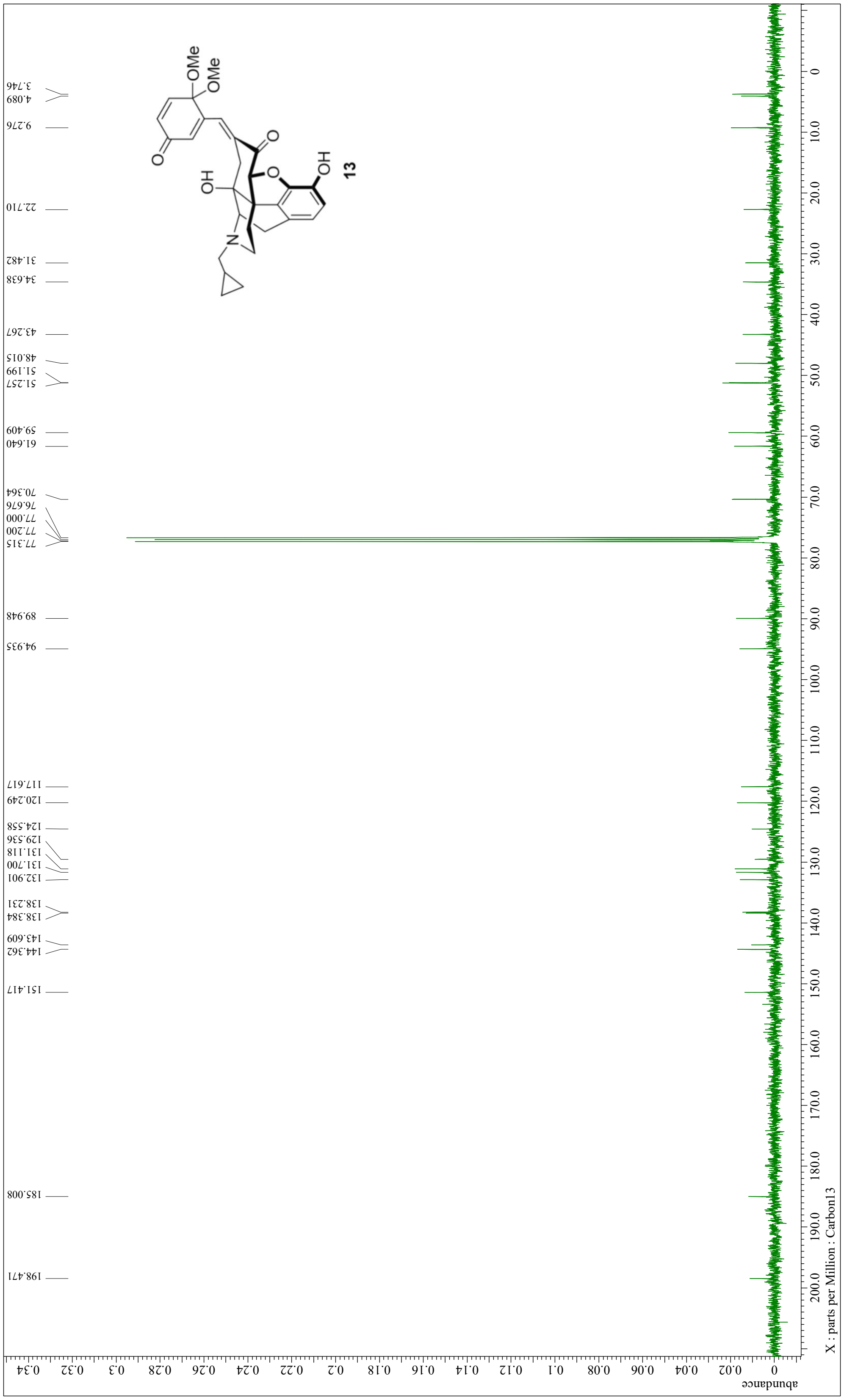

(4R,4aS,7aR,12bS,E)-6-(Cyclohexylmethylene)-3-(cyclopropylmethyl)-4a,9-dihydroxy-2,3,4,4a,5,6-hexahydro-1H-4,12-methanobenzofuro[3,2-e]isoquinolin-7(7aH)-one (**14**)

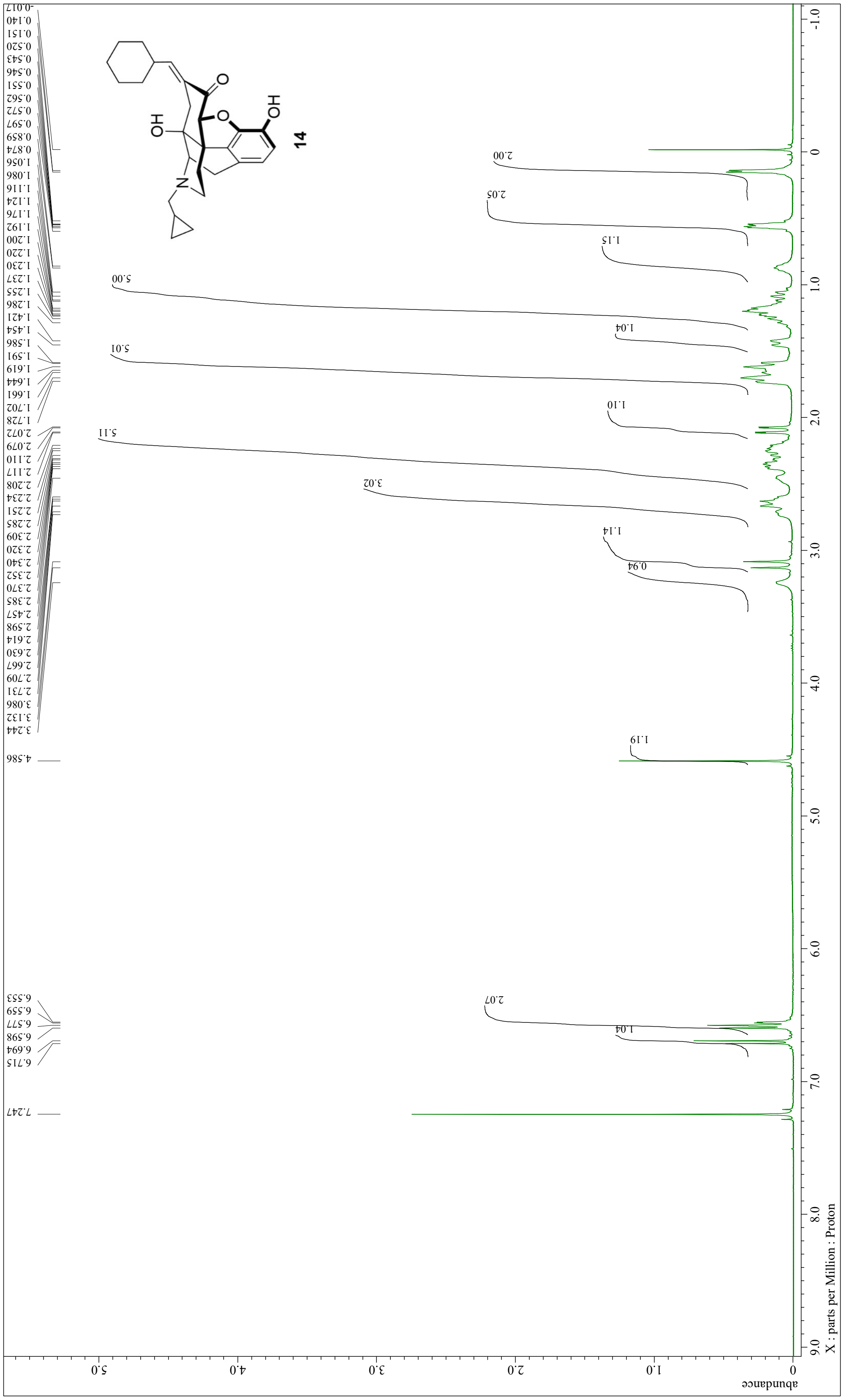

(4R,4aS,7aR,12bS,E)-6-(Cyclohexylmethylene)-3-(cyclopropylmethyl)-4a,9-dihydroxy-2,3,4,4a,5,6-hexahydro-1H-4,12-methanobenzofuro[3,2-e]isoquinolin-7(7aH)-one (**14**)

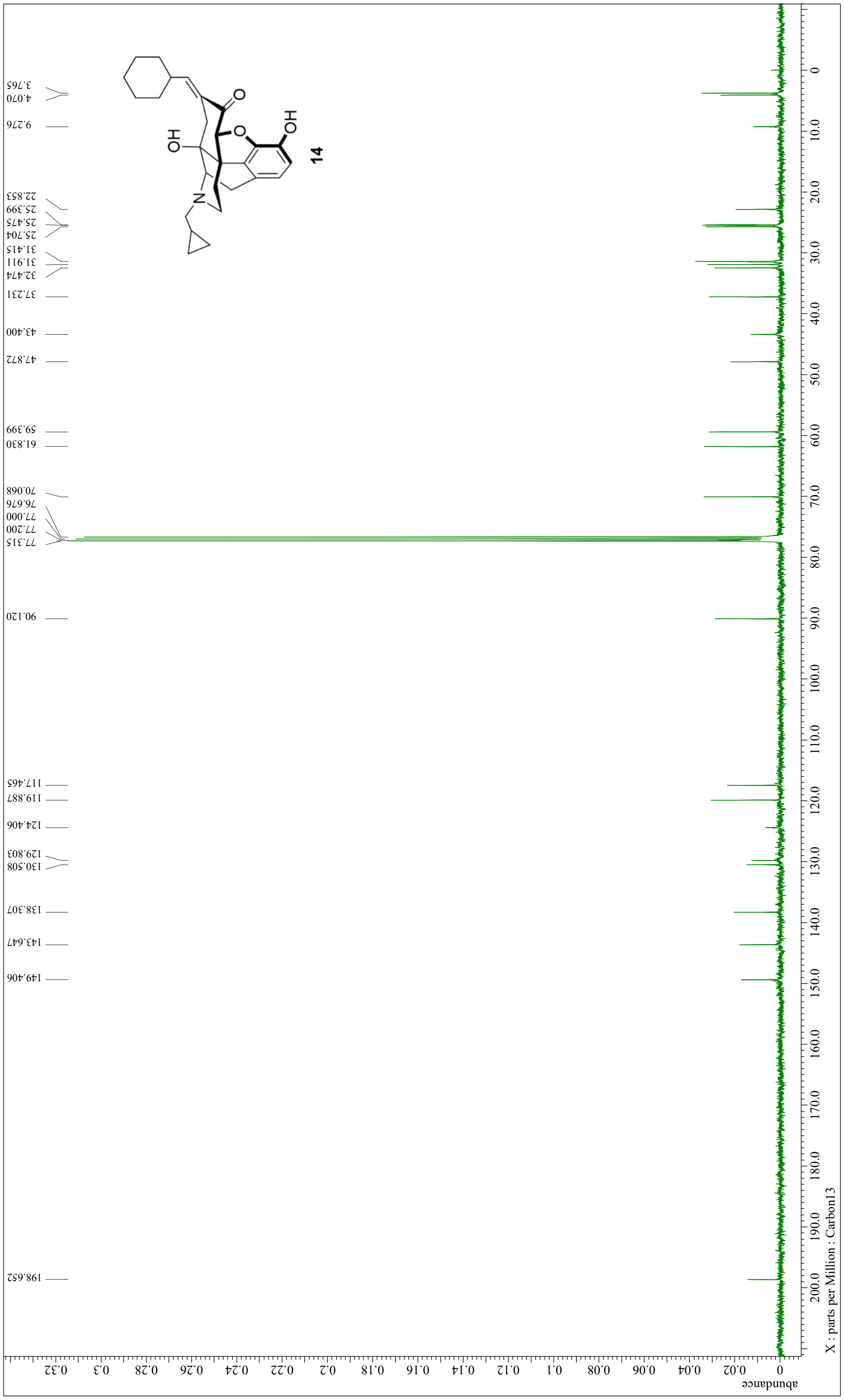

(4R,4aS,7aR,12bS,E)-6-(Cyclobutylmethylene)-3-(cyclopropylmethyl)-4a,9-dihydroxy-2,3,4,4a,5,6-hexahydro-1H-4,12-methanobenzofuro[3,2-e]isoquinolin-7(7aH)-one (**15**)

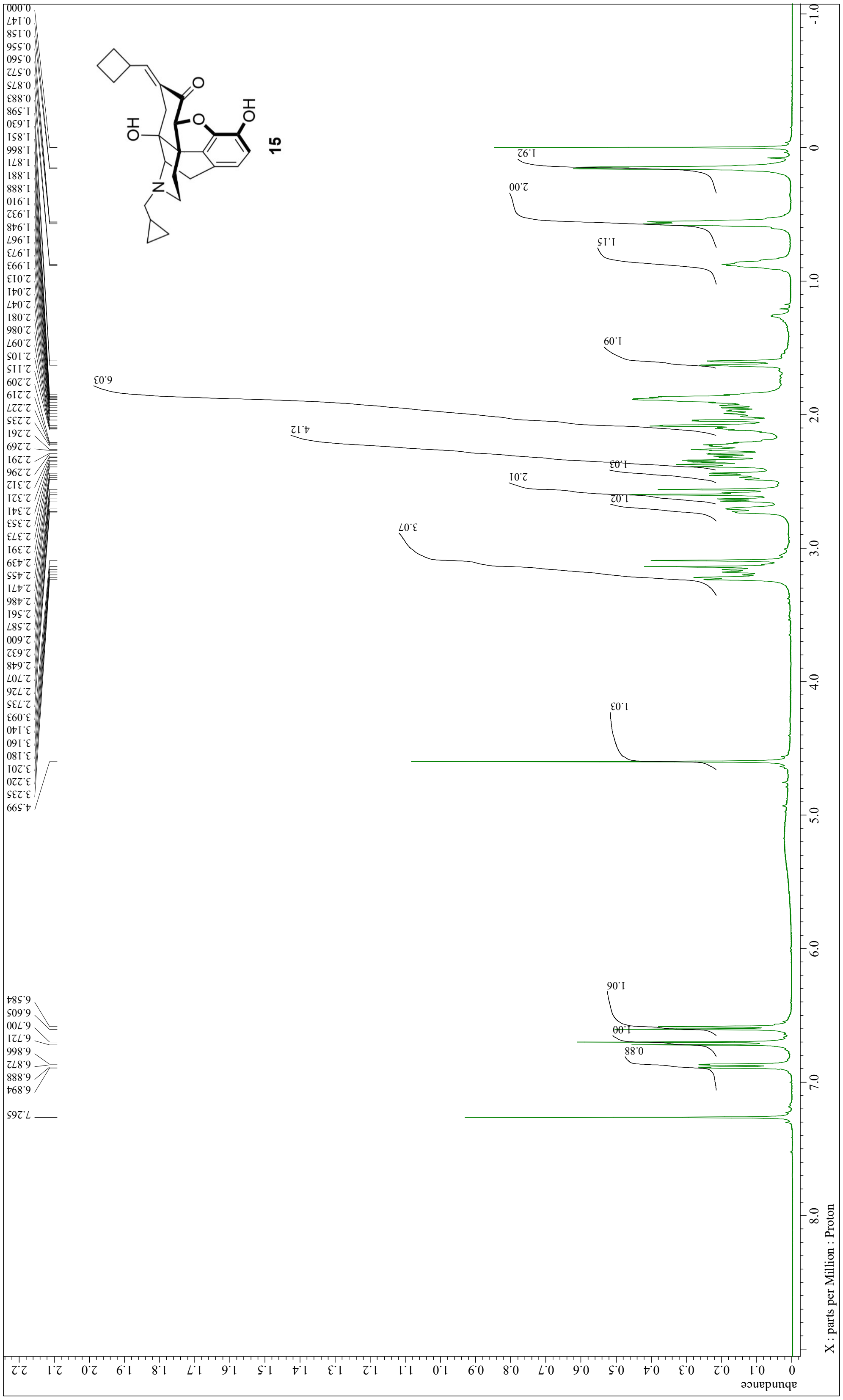

(4R,4aS,7aR,12bS,E)-6-(Cyclobutylmethylene)-3-(cyclopropylmethyl)-4a,9-dihydroxy-2,3,4,4a,5,6-hexahydro-1H-4,12-methanobenzofuro[3,2-e]isoquinolin-7(7aH)-one (**15**)

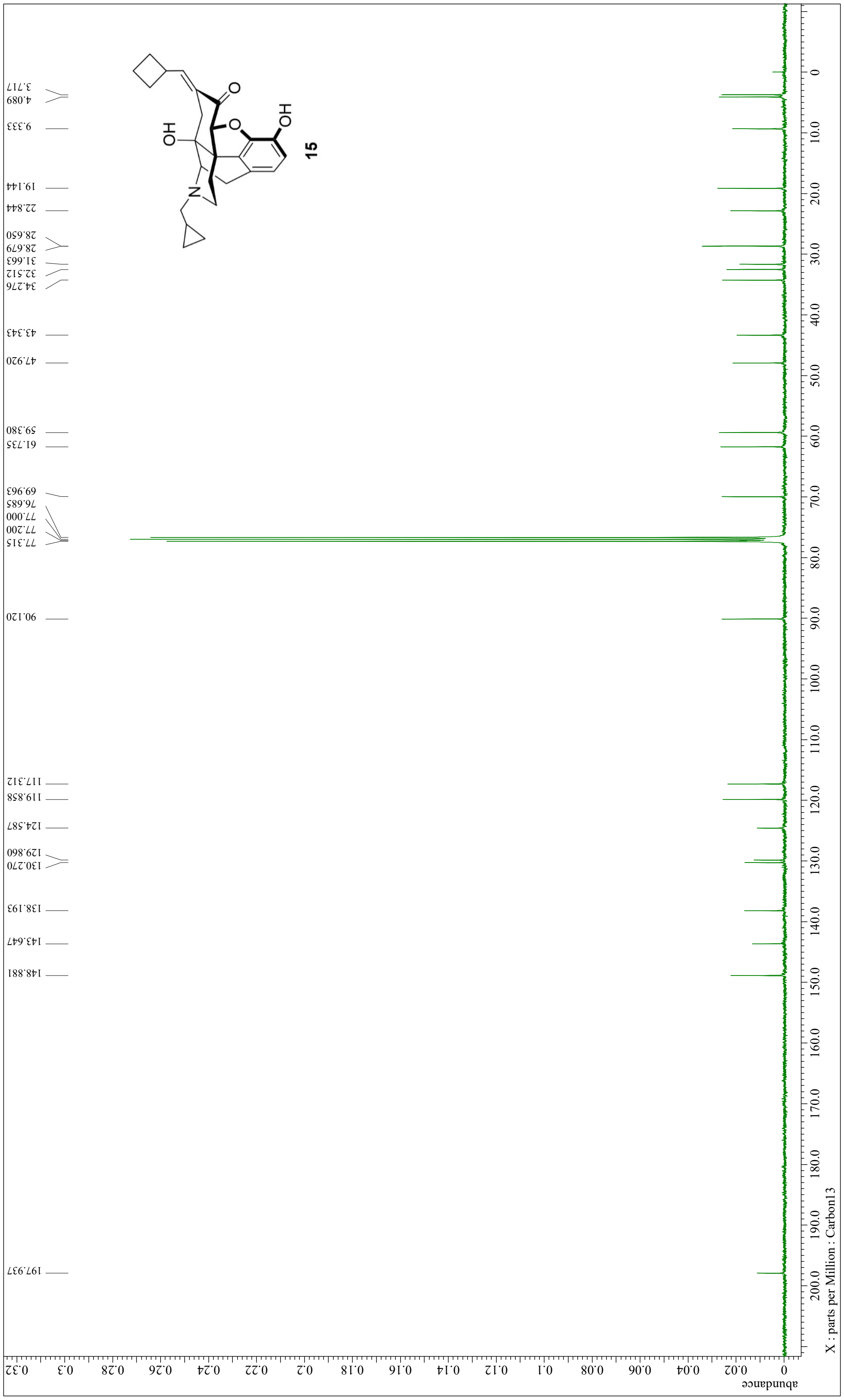

(4R,4aS,7aR,12bS,E)-3-(Cyclopropylmethyl)-6-(cyclopropylmethylene)-4a,9-dihydroxy-2,3,4,4a,5,6-hexahydro-1H-4,12-methanobenzofuro[3,2-e]isoquinolin-7(7aH)-one (**18**)

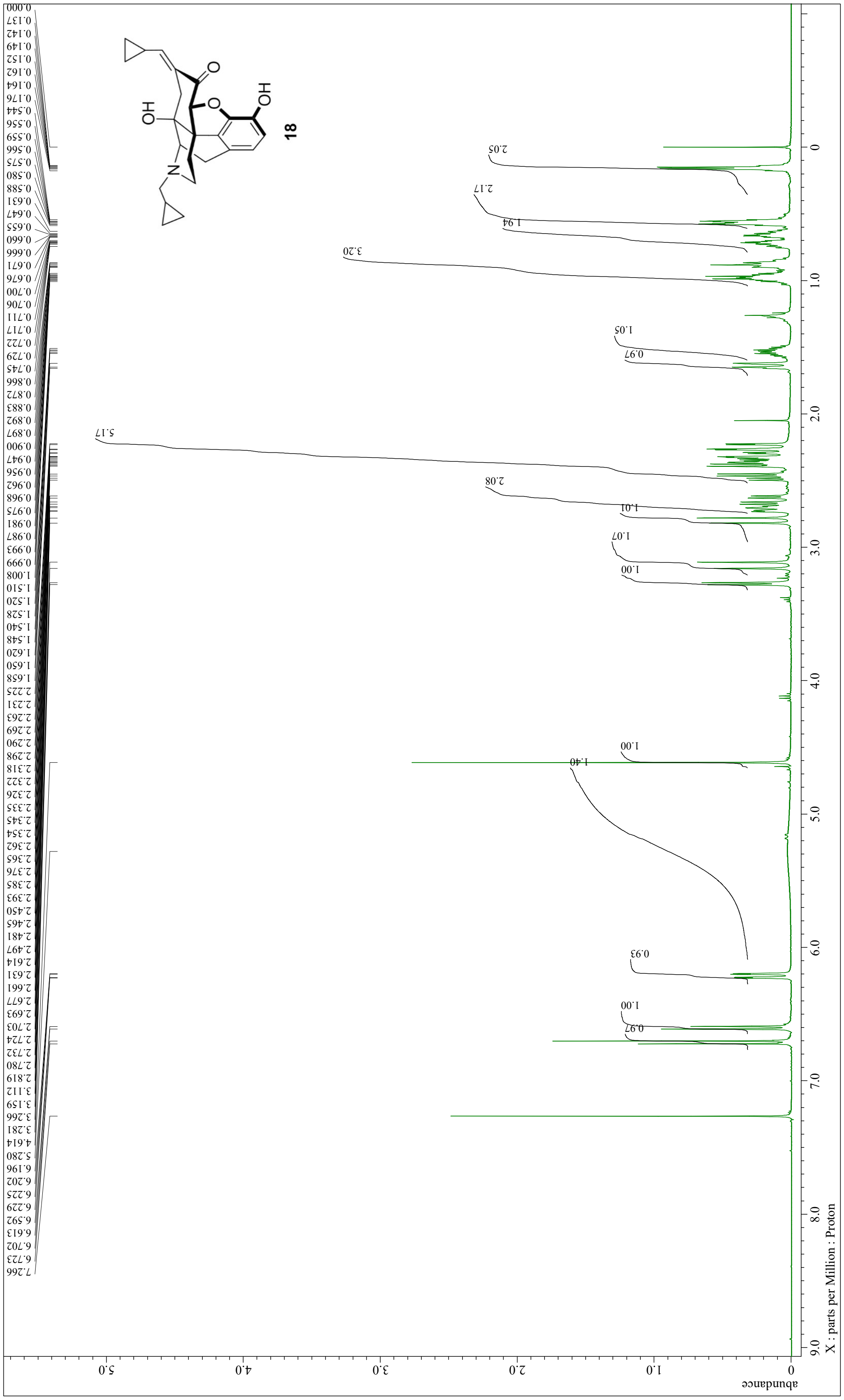

(4R,4aS,7aR,12bS,E)-3-(Cyclopropylmethyl)-6-(cyclopropylmethylene)-4a,9-dihydroxy-2,3,4,4a,5,6-hexahydro-1H-4,12-methanobenzofuro[3,2-e]isoquinolin-7(7aH)-one (**18**)

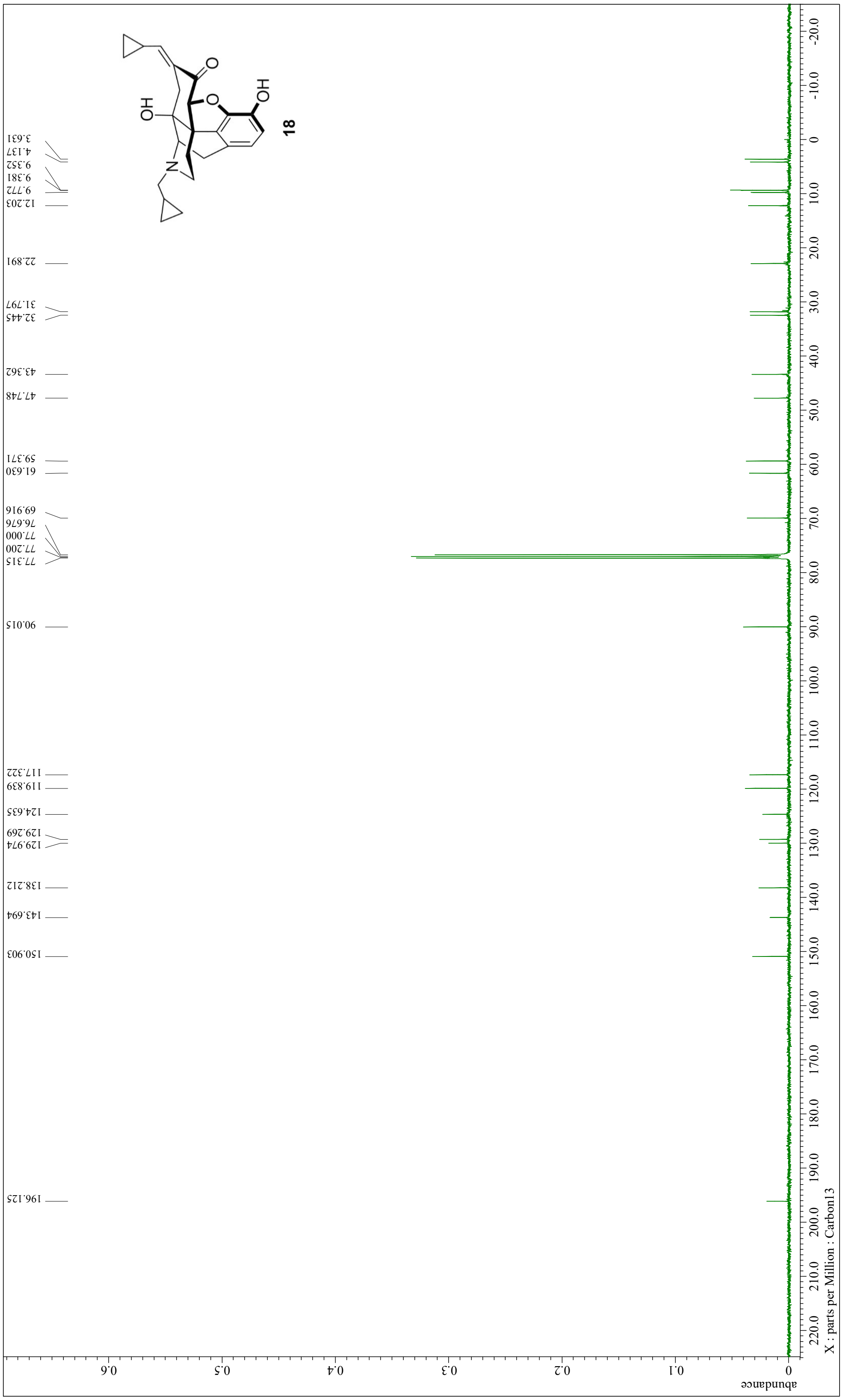

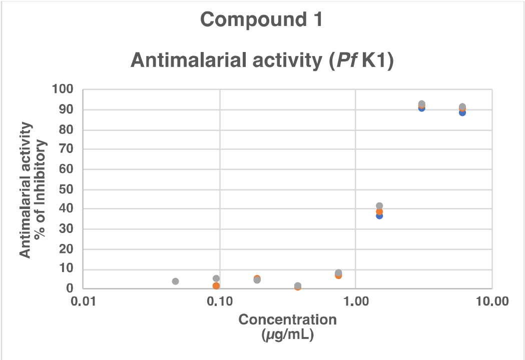
Compound 1
Antimalarial activity (*Pf* K1)
Antimalarial activity
% of Inhibitory
Concentration
(µg/mL)

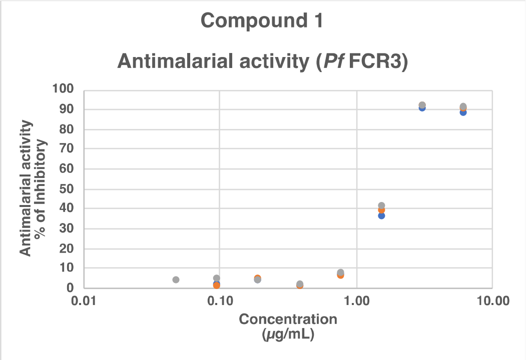
Compound 1
Antimalarial activity (*Pf* FCR3)
Antimalarial activity
% of Inhibitory
Concentration
(µg/mL)

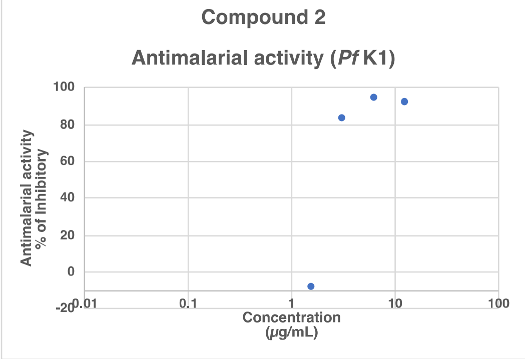
Compound 2
Antimalarial activity (*Pf* K1)
Antimalarial activity
% of Inhibitory
Concentration
(µg/mL)

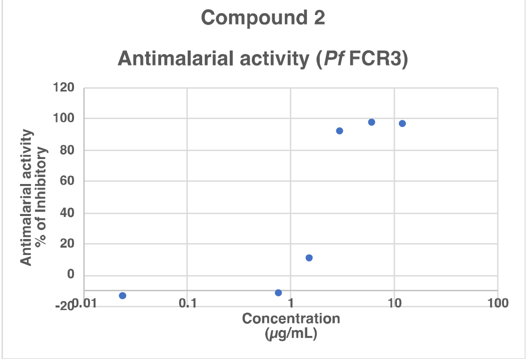
Compound 2
Antimalarial activity (*Pf* FCR3)
Antimalarial activity
% of Inhibitory
Concentration
(µg/mL)

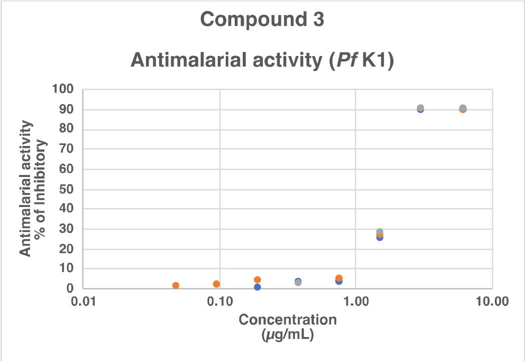
Compound 3
Antimalarial activity (*Pf* K1)
Antimalarial activity
% of Inhibitory
Concentration
(µg/mL)

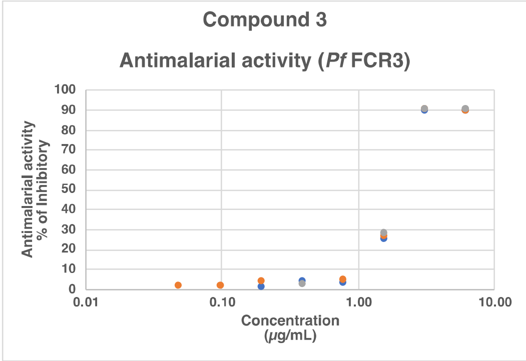
Compound 3
Antimalarial activity (*Pf* FCR3)
Antimalarial activity
% of Inhibitory
Concentration
(µg/mL)

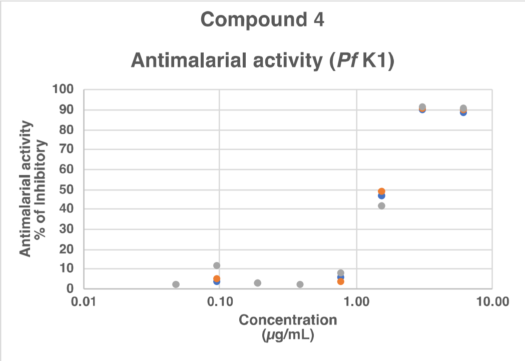
Compound 4
Antimalarial activity (*Pf* K1)
Antimalarial activity
% of Inhibitory
Concentration
(µg/mL)

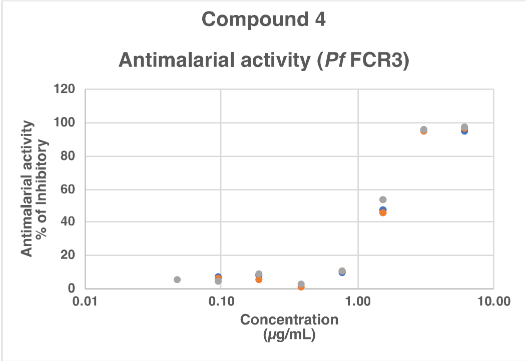
Compound 4
Antimalarial activity (*Pf* FCR3)
Antimalarial activity
% of Inhibitory
Concentration
(µg/mL)

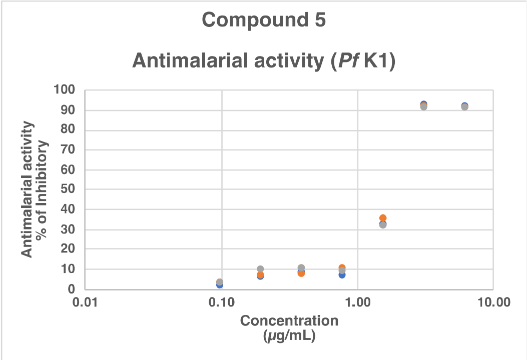
Compound 5
Antimalarial activity (*Pf* K1)
Antimalarial activity
% of Inhibitory
Concentration
(µg/mL)

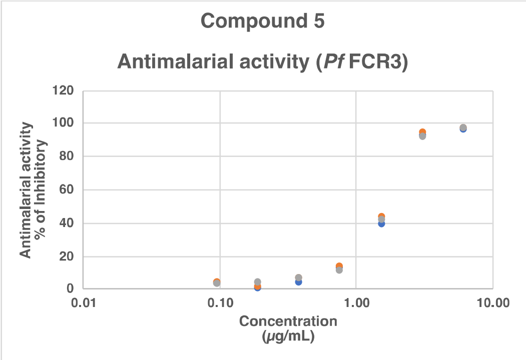
Compound 5
Antimalarial activity (*Pf* FCR3)
Antimalarial activity
% of Inhibitory
Concentration
(µg/mL)

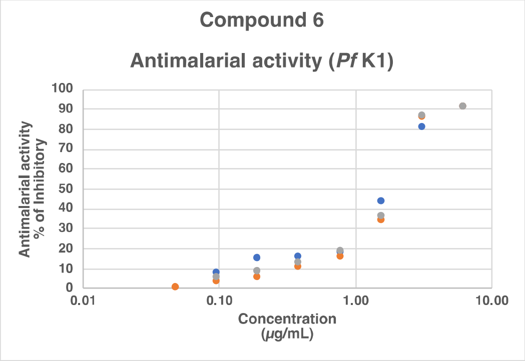
Compound 6
Antimalarial activity (*Pf* K1)
Antimalarial activity
% of Inhibitory
Concentration
(µg/mL)

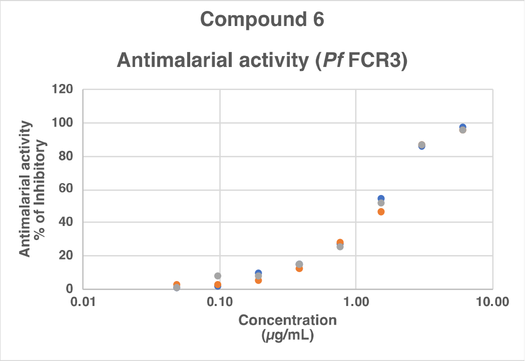
Compound 6
Antimalarial activity (*Pf* FCR3)
Antimalarial activity
% of Inhibitory
Concentration
(µg/mL)

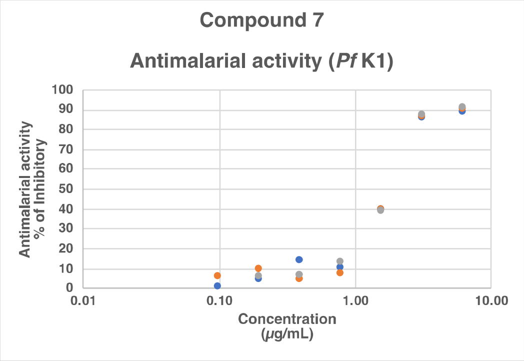
Compound 7
Antimalarial activity (*Pf* K1)
Antimalarial activity
% of Inhibitory
Concentration
(µg/mL)

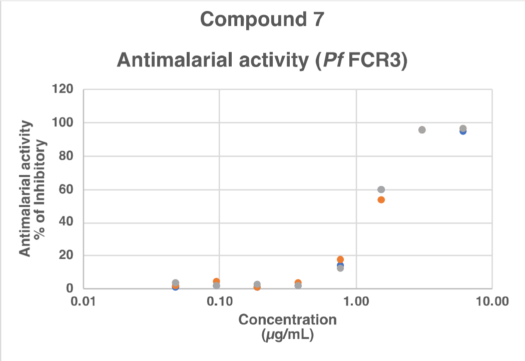
Compound 7
Antimalarial activity (*Pf* FCR3)
Antimalarial activity
% of Inhibitory
Concentration
(µg/mL)

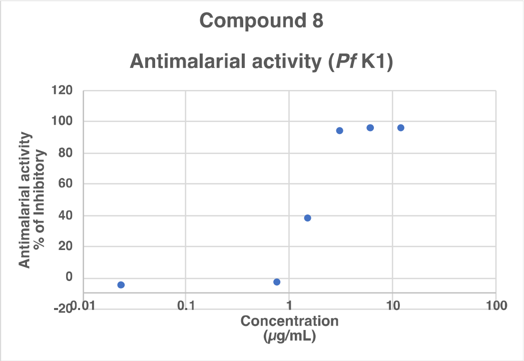
Compound 8
Antimalarial activity (*Pf* K1)
Antimalarial activity
% of Inhibitory
Concentration
(µg/mL)

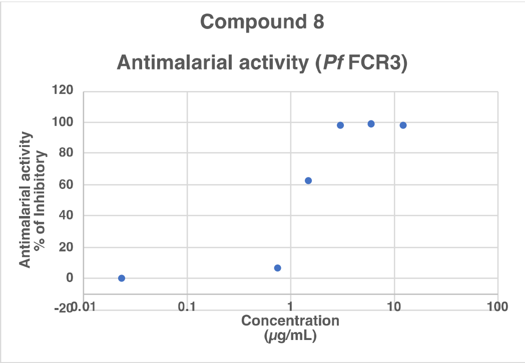
Compound 8
Antimalarial activity (*Pf* FCR3)
Antimalarial activity
% of Inhibitory
Concentration
(µg/mL)

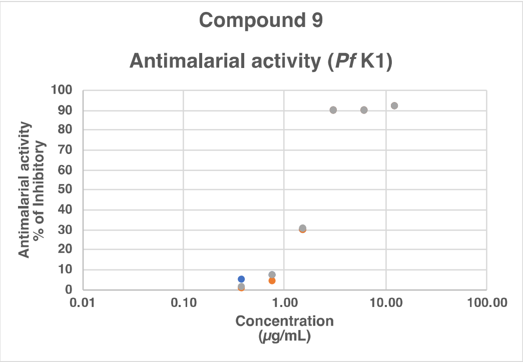
Compound 9
Antimalarial activity (*Pf* K1)
Antimalarial activity
% of Inhibitory
Concentration
(µg/mL)

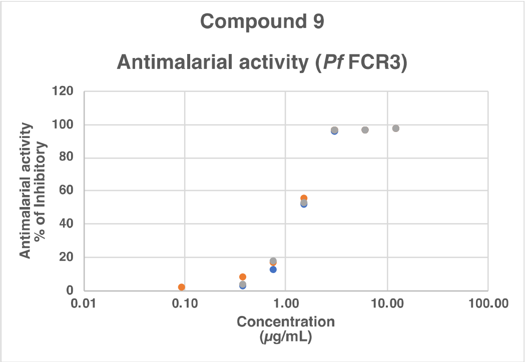
Compound 9
Antimalarial activity (*Pf* FCR3)
Antimalarial activity
% of Inhibitory
Concentration
(µg/mL)

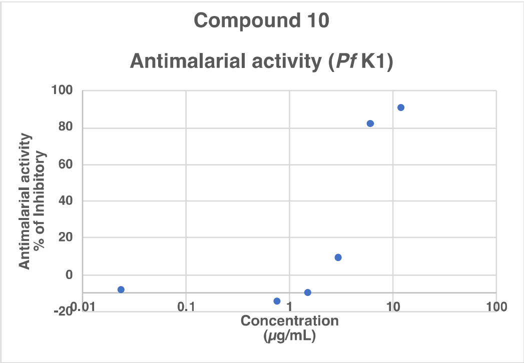
Compound 10
Antimalarial activity (*Pf* K1)
Antimalarial activity
% of Inhibitory
Concentration
(µg/mL)

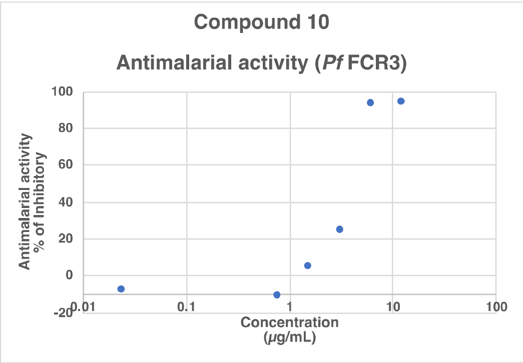
Compound 10
Antimalarial activity (*Pf* FCR3)
Antimalarial activity
% of Inhibitory
Concentration
(µg/mL)

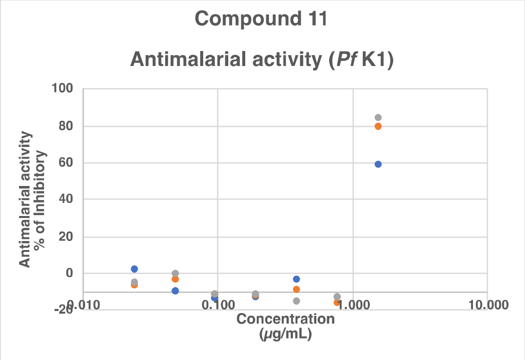
Compound 11
Antimalarial activity (Pf K1)
Antimalarial activity % of Inhibitory
100
80
60
40
20
0
-20
0.010
0.100
1.000
10.000
Concentration (µg/mL)

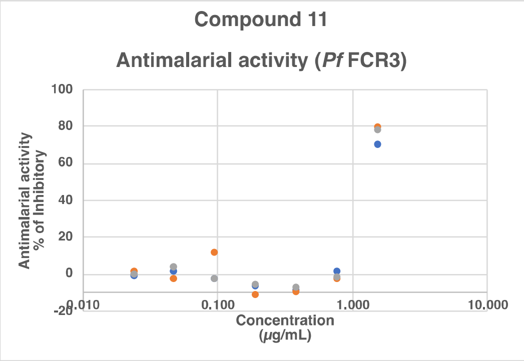
Compound 11
Antimalarial activity (Pf FCR3)
Antimalarial activity % of Inhibitory
100
80
60
40
20
0
-20
0.010
0.100
1.000
10.000
Concentration (µg/mL)

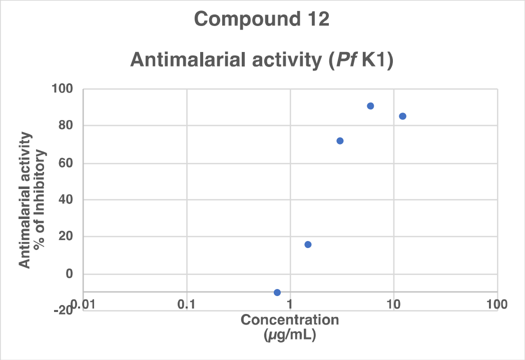
Compound 12
Antimalarial activity (Pf K1)
Antimalarial activity % of Inhibitory
100
80
60
40
20
0
-20
0.01
0.1
1
10
100
Concentration (µg/mL)

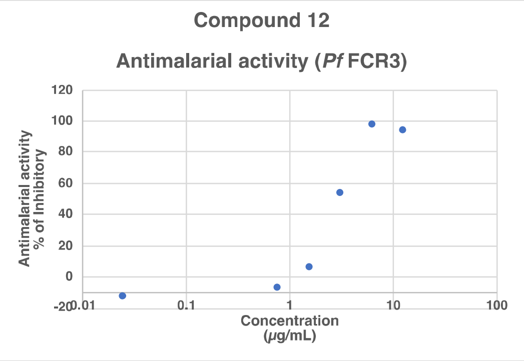
Compound 12
Antimalarial activity (Pf FCR3)
Antimalarial activity % of Inhibitory
120
100
80
60
40
20
0
-20
0.01
0.1
1
10
100
Concentration (µg/mL)

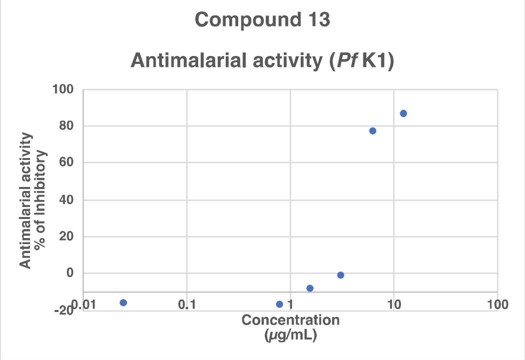
Compound 13
Antimalarial activity (Pf K1)
Antimalarial activity % of Inhibitory
100
80
60
40
20
0
-20
0.01
0.1
1
10
100
Concentration (µg/mL)

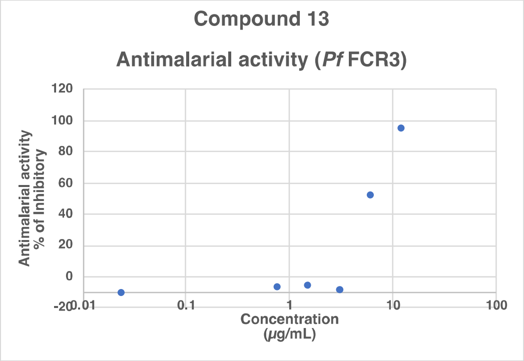
Compound 13
Antimalarial activity (Pf FCR3)
Antimalarial activity % of Inhibitory
120
100
80
60
40
20
0
-20
0.01
0.1
1
10
100
Concentration (µg/mL)

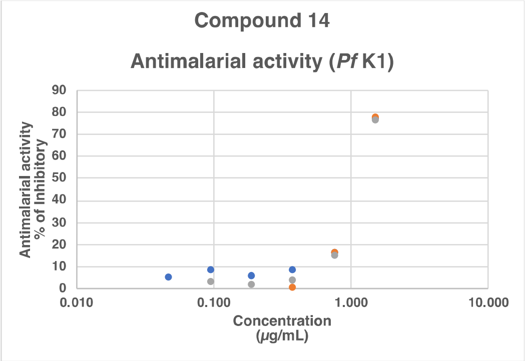
Compound 14
Antimalarial activity (Pf K1)
Antimalarial activity % of Inhibitory
90
80
70
60
50
40
30
20
10
0
0.010
0.100
1.000
10.000
Concentration (µg/mL)

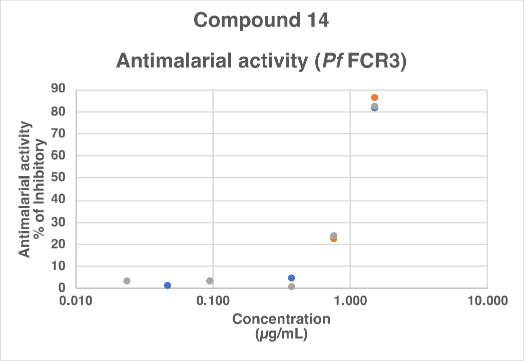
Compound 14
Antimalarial activity (Pf FCR3)
Antimalarial activity % of Inhibitory
90
80
70
60
50
40
30
20
10
0
0.010
0.100
1.000
10.000
Concentration (µg/mL)

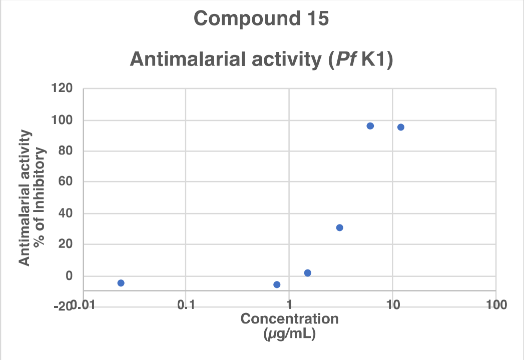
Compound 15
Antimalarial activity (Pf K1)
Antimalarial activity % of Inhibitory
120
100
80
60
40
20
0
-20
0.01
0.1
1
10
100
Concentration (µg/mL)

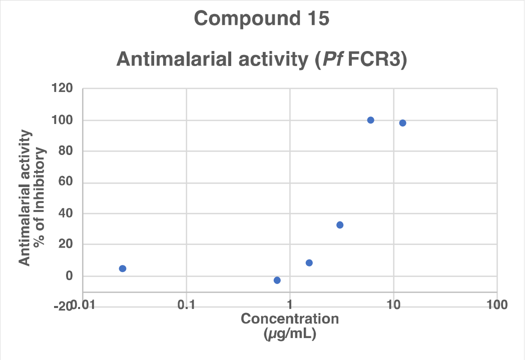
Compound 15
Antimalarial activity (Pf FCR3)
Antimalarial activity % of Inhibitory
120
100
80
60
40
20
0
-20
0.01
0.1
1
10
100
Concentration (µg/mL)

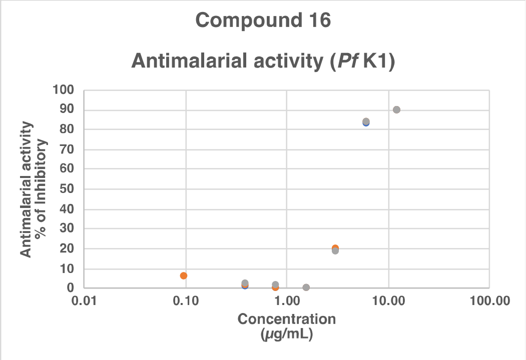
Compound 16
Antimalarial activity (Pf K1)
Antimalarial activity % of Inhibitory
Concentration (µg/mL)

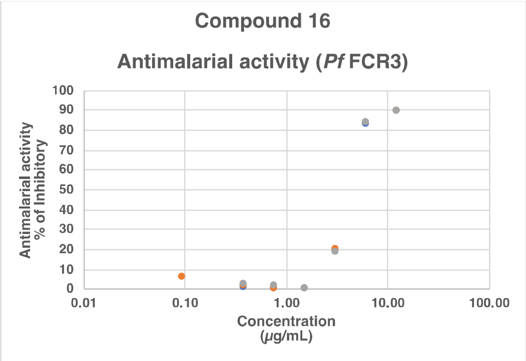
Compound 16
Antimalarial activity (Pf FCR3)
Antimalarial activity % of Inhibitory
Concentration (µg/mL)

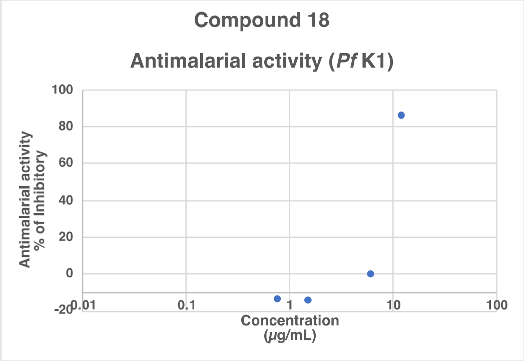
Compound 18
Antimalarial activity (Pf K1)
Antimalarial activity % of Inhibitory
Concentration (µg/mL)

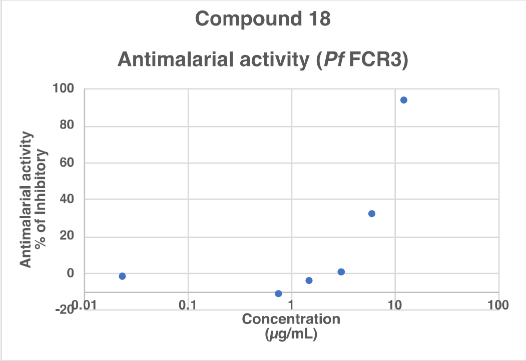
Compound 18
Antimalarial activity (Pf FCR3)
Antimalarial activity % of Inhibitory
Concentration (µg/mL)

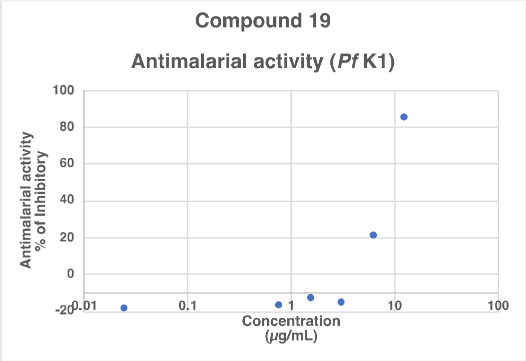
Compound 19
Antimalarial activity (Pf K1)
Antimalarial activity % of Inhibitory
Concentration (µg/mL)

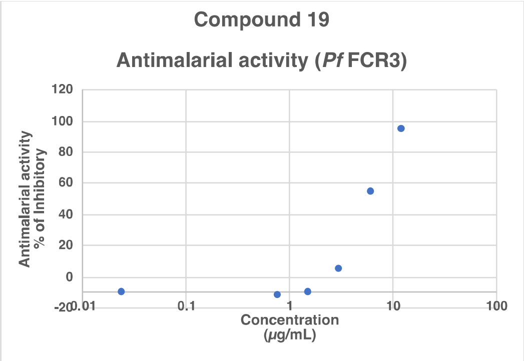
Compound 19
Antimalarial activity (Pf FCR3)
Antimalarial activity % of Inhibitory
Concentration (µg/mL)

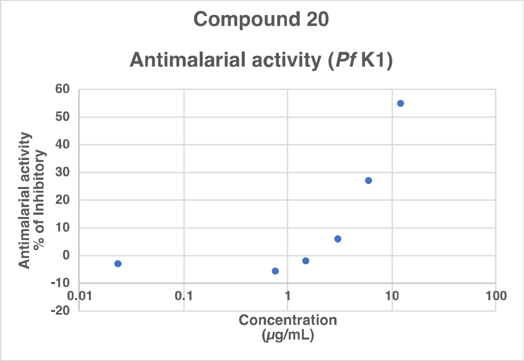
Compound 20
Antimalarial activity (Pf K1)
Antimalarial activity % of Inhibitory
Concentration (µg/mL)

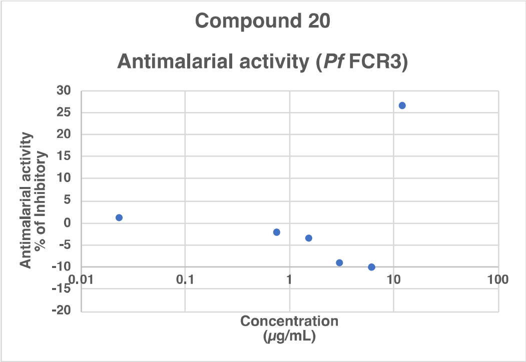
Compound 20
Antimalarial activity (Pf FCR3)
Antimalarial activity % of Inhibitory
Concentration (µg/mL)
